# A candidate antibody drug for prevention of malaria

**DOI:** 10.1038/s41591-023-02659-z

**Published:** 2024-01-02

**Authors:** Katherine L. Williams, Steve Guerrero, Yevel Flores-Garcia, Dongkyoon Kim, Kevin S. Williamson, Christine Siska, Pauline Smidt, Sofia Z. Jepson, Kan Li, S. Moses Dennison, Shamika Mathis-Torres, Xiaomu Chen, Ulrike Wille-Reece, Randall S. MacGill, Michael Walker, Erik Jongert, C. Richter King, Christian Ockenhouse, Jacob Glanville, James E. Moon, Jason A. Regules, Yann Chong Tan, Guy Cavet, Shaun M. Lippow, William H. Robinson, Sheetij Dutta, Georgia D. Tomaras, Fidel Zavala, Randal R. Ketchem, Daniel E. Emerling

**Affiliations:** 1 Atreca, Inc., San Carlos, CA USA; 2 Department of Molecular Microbiology and Immunology, Malaria Research Institute, Johns Hopkins Bloomberg School of Public Health, Baltimore, MD USA; 3 Initium Therapeutics, Inc., Natick, MA USA; 4 Just – Evotec Biologics, Seattle, WA USA; 5 Duke Center for Human Systems Immunology, Department of Surgery, Duke University, Durham, NC USA; 6 BioNTech US, Inc., Cambridge, MA USA; 7 PATH Center for Vaccine Innovation and Access, Washington DC, USA; 8 Walker Bioscience, Carlsbad, CA USA; 9 GSK, Rixensart, Belgium; 10 Centivax, Inc., South San Francisco, CA USA; 11 Center for Enabling Capabilities, Walter Reed Army Institute of Research, Silver Spring, MD USA; 12 Walter Reed Army Institute of Research, Silver Spring, MD USA; 13 Nuevocor Pte. Ltd, Singapore, Singapore; 14 Paramune, Inc., San Carlos, CA USA; 15 Division of Immunology and Rheumatology, Department of Medicine, Stanford University School of Medicine, Stanford, CA USA; 16 Departments of Immunology, Molecular Genetics and Microbiology, Human Vaccine Institute, Duke University, Durham, NC USA

**Keywords:** Antibody therapy, Immune evasion, Protein vaccines, Antibody generation, Malaria

## Abstract

Over 75% of malaria-attributable deaths occur in children under the age of 5 years. However, the first malaria vaccine recommended by the World Health Organization (WHO) for pediatric use, RTS,S/AS01 (Mosquirix), has modest efficacy. Complementary strategies, including monoclonal antibodies, will be important in efforts to eradicate malaria. Here we characterize the circulating B cell repertoires of 45 RTS,S/AS01 vaccinees and discover monoclonal antibodies for development as potential therapeutics. We generated >28,000 antibody sequences and tested 481 antibodies for binding activity and 125 antibodies for antimalaria activity in vivo. Through these analyses we identified correlations suggesting that sequences in *Plasmodium falciparum* circumsporozoite protein, the target antigen in RTS,S/AS01, may induce immunodominant antibody responses that limit more protective, but subdominant, responses. Using binding studies, mouse malaria models, biomanufacturing assessments and protein stability assays, we selected AB-000224 and AB-007088 for advancement as a clinical lead and backup. We engineered the variable domains (Fv) of both antibodies to enable low-cost manufacturing at scale for distribution to pediatric populations, in alignment with WHO’s preferred product guidelines. The engineered clone with the optimal manufacturing and drug property profile, MAM01, was advanced into clinical development.

## Abstract (web summary)

Isolation and optimization of antibodies targeting the malaria parasite may offer the potential for immediate protection as a prophylactic intervention to prevent severe disease.

## Main

Malaria is a mosquito-borne, parasitic disease endemic in regions impacting over 1.5 billion people in Asia, the Americas, the Middle East and Africa. More than 247 million malaria cases and 619,000 malaria-related deaths were reported in 2021 (ref. ^1^), with 76.8% of these deaths occurring in children under the age of 5 years. WHO has determined that reduction of morbidity and mortality in infants and children due to *Plasmodium falciparum* is among the most urgent priorities in combatting malaria^2^. Although vaccination has been a key tool in control and eradication of other infectious diseases, the development of a vaccine for malaria has been a 50-year challenge^3^. The first vaccine recommended for use by WHO, RTS,S/AS01 (Mosquirix), targets the circumsporozoite (CSP) protein of *P. falciparum* (PfCSP), the malaria species primarily responsible for mortality in Africa^1,3^. Recently a second vaccine, R21/Matrix M, based on the same CSP-derived antigen as RTS,S^4^, was also recommended for use^5^. Immunization with CSP antigen induces anti-CSP antibodies that act by binding to sporozoites, the infective form of the malaria parasite introduced by mosquito bite, and by inhibiting their initial infection of liver cells^6,7^. However, vaccine efficacy against clinical malaria induced by RTS,S/AS01 in children is limited to 45% after the first dose and wanes to 36% over 4 years of follow-up^8^. Chemoprevention-based prophylactics are an alternative to vaccines, but drug resistance and complex drug regimens that lead to poor adherence^1^ limit their utility.

Longlasting antimalaria monoclonal antibody (mAb) prophylaxis could complement these existing prevention strategies by providing immediate and more stable serum antibody levels and limiting adherence concerns^2^. However, the potential high cost of antibody drugs can be a barrier to access in low-to-middle-income countries (LMICs) and administration to pediatric populations requires small-volume doses of highly concentrated drugs amenable to low-viscosity formulations for administration via small needles. For these reasons, the WHO guidelines for monoclonal antibody use in malaria prevention recommend early consideration of key factors that contribute to the cost of goods, including manufacturing properties, and to drug viscosity, including biophysical properties such as protein–protein interactions, protein aggregation and protein conformational and colloidal stability^9^.

Recent reports show that treatment with mAbs^10^ can completely prevent malaria following controlled human infection^11^ and provide 88% efficacy (prevention of infection) for 6 months in endemic regions^12^, although limited details have been published about the biophysical properties consistent with manufacturing or cost-effective administration of these antibodies to pediatric populations living in LMICs. Two mAbs already tested in clinical trials, L9 (ref. ^13^) and CIS43 (refs. ^11,12^), were isolated from B cells of vaccinees immunized with whole sporozoites and can prevent malaria infection by targeting specific epitopes on CSP. CSP comprises three main domains: (1) an N terminus; (2) a central repeat region composed of multiple (25–40) NANP tetrapeptides (‘major repeat’) interspersed with an NPDP tetrapeptide and two to four NVDP (‘minor repeat’) tetrapeptides; and (3) a C-terminal domain^14,15^. L9 and CIS43 preferentially bind epitopes containing, respectively, NPNV^16^ in the minor-repeat region and DPNA^17^ in the ‘junctional region’ (JR) that links the N-terminal and repeat domains. However, both mAbs can promiscuously bind NPNA epitopes in the central repeat region^16^. These NPNA repeats are conserved across all Pf strains^18,19^ and are the only tetrapeptides included in RTS,S. A third mAb, AB-000317 (ref. ^20^), preferentially binds NPNA epitopes. Despite its potent inhibitory activity in vivo^16,20,21^, AB-000317 was not advanced into clinical development due to unpublished evidence of suboptimal cell line expression levels and human tissue cross-reactivity.

Here we report on the discovery of >50 preclinically protective antibody lineages (including the original discovery of AB-000317) by sequencing plasmablast (PB) repertoires from 45 RTS,S/AS01 vaccinees. In conducting these studies we observed that, for many vaccinees, antibodies expressed by these circulating PBs were insufficient to protect against malaria challenge. Subsequent investigation uncovered an inverse association between vaccinee protection status and CSP reactivity of antibodies expressed from their PB repertoires, suggesting that RTS,S/AS01 vaccination may induce immunodominant anti-CSP responses that do not contribute to protective immunity. Given the published support for prophylaxis with mAbs as a strategy against malaria^11–13^, and recent advances using mAbs as therapeutics and prophylactics against infectious diseases^22^, we aimed to select and engineer (Supplementary Fig. 1) a potent and longlasting antibody drug with biophysical properties amenable for cost-effective manufacturing and dosing in pediatric populations^9,23^.

## Results

### Initial mAb library includes representative clones from dominant PB lineages

**Extended Data Fig. 1:**
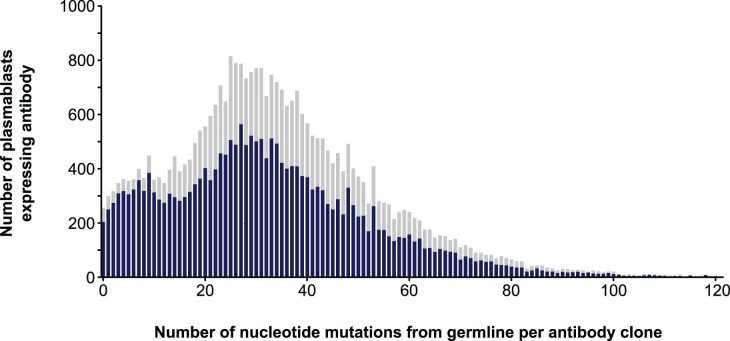
Levels of SHM among antibody sequences. Histogram of the number of nucleotide mutations from germline (SHM) of combined IgG heavy and light chains from blood PBs collected 7 days after administration of the third dose (blue, *n* = 22,319) or fourth dose (grey, *n* = 10,629) of RTS,S. Source data

Peripheral blood mononuclear cells (PBMCs) were obtained from vaccinees enrolled in a phase 2a clinical trial^24^ (NCT01857869) designed to evaluate either three full doses of RTS,S/AS01_B_ 1 month apart (012M; *n* = 15) or two full doses 1 month apart and a smaller (one-fifth, ‘fractional’) dose 6 months later (Fx017M, *n* = 30). Vaccinees were challenged with malaria in a controlled human malaria infection (CHMI) model 3 weeks after the third dose. A subset (*n* = 19) received a fourth dose 8 months after the third dose and were subsequently challenged a second time with malaria (Supplementary Fig. 2). PBs were isolated from PBMCs collected 7 days post third (P3D; *n* = 22,319 PB) and post fourth doses (P4D; *n* = 10,629 PB; Supplementary Table 1) and were used to generate natively paired heavy- and light-chain IgG sequences using our Immune Repertoire Capture sequencing platform. Almost all (99.2%) of the antibody sequences were divergent from inferred germline precursor sequences (Extended Data Fig. 1 and Methods). Consistent with previous malaria studies^17,25–28^, specific germline heavy- and light-chain genes and pairings, including IGHV3-30/33, KV1-5, KV3-20 and LV1-40, were observed frequently in the dataset (Supplementary Figs. 3–5). No significant associations were observed between vaccinee protection status or between vaccinee dose group and multiple IgG sequences and repertoire features (Supplementary Figs. 3–7).

**Fig. 1:**
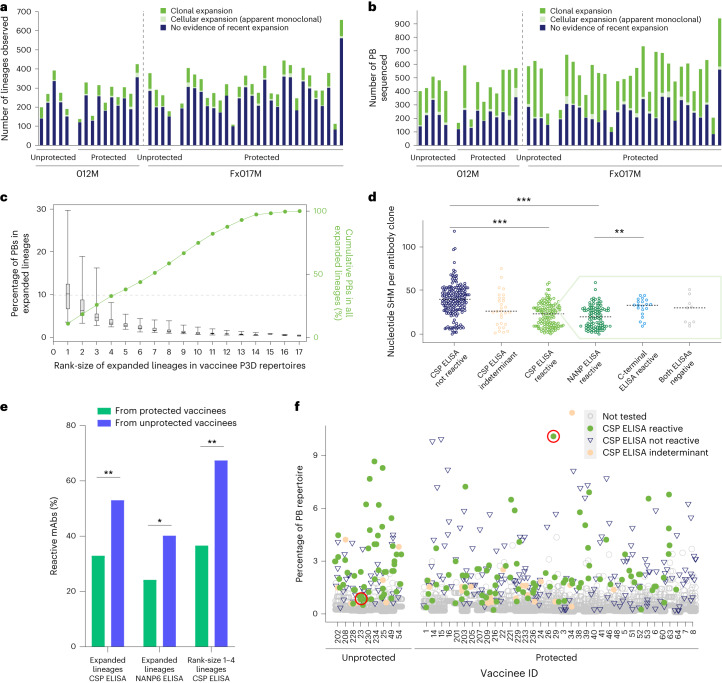
CSP-reactive lineages from blood PBs following the third dose of RTS,S. **a**,**b**, IgG lineages for each vaccinee (bars, *n* = 45) that are clonally expanded (that is, have two or more distinct nucleotide clones; green), that consist of two or more identical nucleotide clones (gray) or that contain only one observed PB (blue) are shown by either number of lineages (**a**) or number of PBs per vaccinee (**b**). **c**, By vaccinee (*n* = 45), the size of each expanded lineage was calculated by dividing the number of PBs in that lineage (2–84 PBs per lineage) by the number in each vaccinee’s P3D-expanded lineage repertoire (37–492 PBs per vaccinee) and then assigning a rank-size. Boxes indicate interquartile ranges, lines within boxes are medians and whiskers represent minimum and maximum across vaccinees for each rank-size (44–1,246 PBs per rank, 22–301 lineages per rank). Dotted line indicates that the top four rank-size lineages contain 33% of PBs in all P3D-expanded lineages (*n* = 11,478 PBs, 2,662 lineages). **d**–**e**, ELISA reactivity, SHM levels and vaccinee protection status of mAbs from expanded lineages (*n* = 349). **d**, Number of nucleotide mutations from germline (SHM) for mAbs that are not reactive (dark blue, *n* = 185), show an indeterminant, weak signal (orange, *n* = 29) or are reactive (light green, *n* = 135) in a CSP ELISA. Domain specificity for CSP-reactive mAbs is shown in the box with a dashed green outline. Monoclonal antibodies reactive by ELISA to the NANP6 repeat-region peptide (dark green, *n* = 98), to the C-terminal region peptide (Pfs16, royal blue, *n* = 20) or not reactive in either peptide ELISA (gray, *n* = 9). Lines represent median values, ***P < 0.0001, **P < 0.001, unpaired two-tailed Mann–Whitney test. CSP-reactive mAbs that were not tested in peptide ELISAs (*n* = 8) are not shown. **e**, Percentage of tested antibodies from expanded lineages that originated from protected (green, *n* = 36) and unprotected (blue, *n* = 9) vaccinees that are CSP-reactive (82 out of 249, and 53 out of 100 mAbs, respectively), repeat-region NANP6 peptide-reactive (59 out of 244, and 39 out of 97 mAbs, respectively) and the subset from only dominant rank-size one to four lineages that are CSP-reactive (52 out of 142, and 31 out of 46 mAbs, respectively); **P < 0.001, *P < 0.01, two-tailed Fisher’s exact test. **f**, For each vaccinee (*x* axis), each symbol indicates a single lineage. The lineages (*n* = 369) from which a clone was selected for testing are indicated by CSP reactivity: CSP-reactive (green dots, *n* = 139), indeterminant (orange dots, *n* = 29) or not reactive (royal blue triangles, *n* = 201). All lineages that were not tested are shown (gray circles, *n* = 13,134; 2,313 expanded and 10,821 single-PB lineages). Protected vaccinees have a lower ratio of CSP-reactive versus nonreactive lineages than unprotected vaccinees (bootstrap analysis, *P* = 0.0011). Red circles indicate the two lineages containing the amino acid sequence of AB-000317.

**Extended Data Fig. 2:**
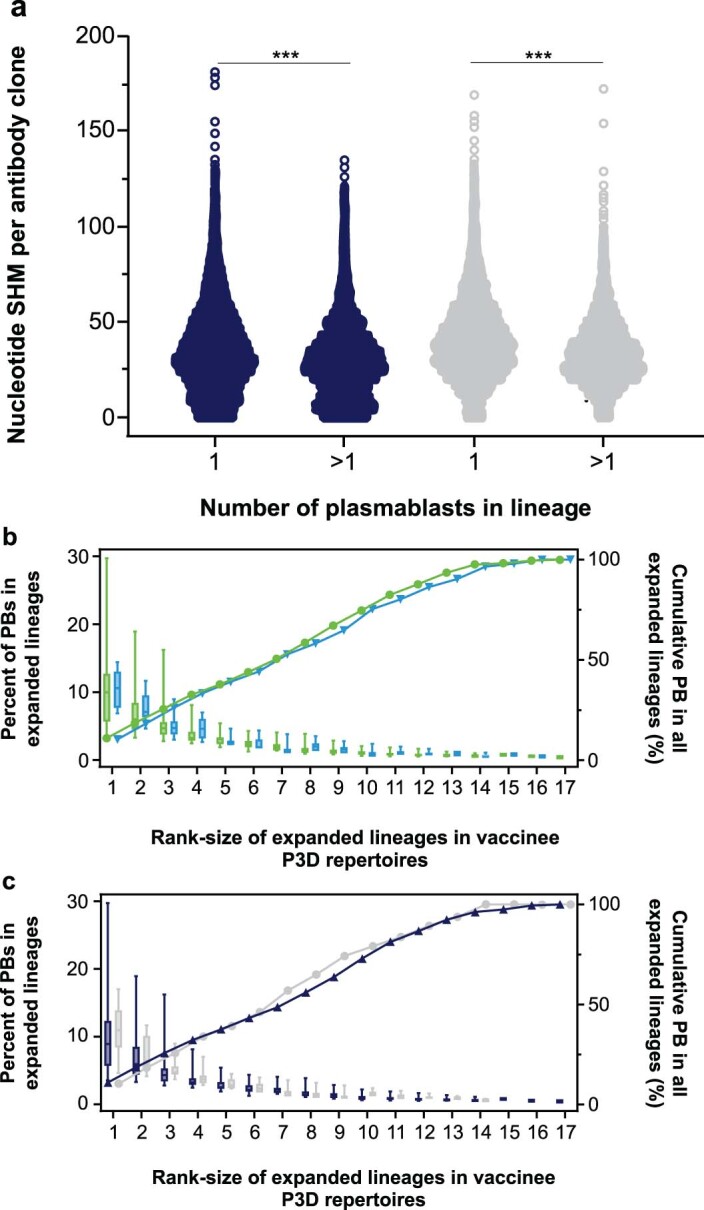
SHM and size distribution of expanded PB IgG lineages. **a**, Range of nucleotide mutations from germline (SHM) in heavy plus light chain for antibodies in expanded lineages (>1 PB) compared to antibodies in lineages that lack evidence of recent expansion (1 PB). PBs were collected 7 days after administration of the third dose (blue, *n* = 11,478 and *n* = 10,841, respectively) or fourth dose (grey, *n* = 5,538 and *n* = 5,091, respectively) of RTS,S, ***P < 0.0001 unpaired two-tailed Mann–Whitney test. **b**, **c**, By vaccinee, the size of each expanded lineage was calculated by dividing the number of PBs in that lineage by the number of PBs in all expanded lineages within each repertoire and then assigning a rank-size. Box and whisker plots represent the distribution of lineage sizes across vaccinees for each rank-size from, **b**, protected (green, *n* = 36) versus unprotected (blue, *n* = 9) vaccinees, and, **c**, from vaccinees administered a third standard dose (012 M, grey, *n* = 15) versus vaccinees administered a delayed fractional dose (Fx017M, blue, *n* = 30) of RTS,S. Boxes indicate interquartile ranges, lines within boxes are medians, and whiskers represent minimum and maximum across vaccinees.

P3D and P4D PBs that were probably derived from a common progenitor B cell clone were grouped into Ig lineages (*n* = 18,980; Methods). Lineage size ranged from 1 to 84 PBs (P3D) and from 1 to 93 PBs (P4D). Because PBs have a short half-life in blood^29^ (reviewed in refs. ^30,31^) and were isolated from a small volume of blood (~10 ml), detection of lineages with two or more PBs probably indicates recent cellular expansion in lymphoid organs. One-fifth of lineages contained at least two PBs (‘expanded lineages’, 19.4%, *n* = 3,684 lineages; Fig. 1a). Consistent with antigen-driven selection pressure following vaccination, most of these lineages were clonally expanded and included two or more distinct antibody sequences (Fig. 1b). Several lineages had clonal representatives that were observed after both the third and fourth immunization (‘recalled lineages’; 4.1–26.6% of vaccinee P3D-expanded lineages). Furthermore, we observed that many of these expanded lineages also showed evidence of sequence convergence between two or more vaccinees (7.3–46.7% of P3D-expanded lineages; Methods). Not surprisingly, lineages with only a single observed PB in P3D repertoires (*n* = 10,841) had significantly lower rates of convergence (2.0–13.8%) and recall (1.2–18.6%) than expanded lineages (*P* < 0.0001 and *P* < 0.001, respectively, Wilcoxon matched-pairs, two-tailed test) and had higher levels of somatic hypermutation (SHM; Extended Data Fig. 2a). Thus, to increase the likelihood of identifying antibodies derived against the RTS,S antigen we mainly focused subsequent analyses on expanded lineages (Fig. 1b).

We hypothesized that lineages with the largest number of PBs per vaccinee (‘dominant lineages’) were more likely to target the vaccine because they had outcompeted other PB lineages for antigen binding and/or T cell help in lymphoid organs. Thus, for each vaccinee, expanded P3D lineages were rank ordered by size (‘rank-size’). The sum of PBs in the top four rank-sized lineages constituted 17–100% of each vaccinee’s P3D-expanded lineage repertoire and 33% of PBs in all of the vaccinees’ P3D-expanded lineages (Fig. 1c). Because this pattern of PB distribution was consistent across vaccinee protection status and dose regimens (Extended Data Fig. 2b,c), we generated a mAb screening library that enriched for the dominant P3D lineages of both protected and unprotected vaccinees.

### CSP-reactive mAbs have less SHM and are more predominant in unprotected vaccinees

We selected a library of 369 clones, each representing a unique P3D lineage, for testing (Methods and Supplementary Fig. 8a). This library included almost all (96%) of the largest lineages (rank-size 1); approximately half (56%) of the second, third and fourth rank-size lineages; a minor subset (6.9%) of smaller rank-sized lineages (rank-size five or more); and a few single-PB lineages (0.18% of 10,841 single-cell lineages). All mAbs (*n* = 369) were screened in a CSP ELISA (Fig. 1d and Supplementary Fig. 8b), and a subset (*n* = 130) were screened against the other RTS,S component, hepatitis B surface antigen (HBsAg; Supplementary Fig. 8c). Of the mAbs screened in both assays, 52% (67 out of 130) were reactive to either CSP (*n* = 28) or HBsAg (*n* = 38) and one additional antibody was reactive in both assays. In total, 38% (139 out of 369) were reactive to CSP and binding for an additional 29 mAbs was indeterminate. Of the CSP-reactive mAbs, 73% (102 out of 139, including AB-000317) bound the NANP6 peptide and 14% (20 out of 139) bound peptides from the C-terminal region (Fig. 1d and Supplementary Table 2).

Given that expanded lineages were more likely to show evidence of convergence and recall compared with single PBs, we tested whether those same features were associated with CSP reactivity. Indeed, mAbs from convergent or recalled lineages were more likely to bind to CSP compared with those from lineages observed in only one vaccinee (54% (55 out of 102) versus 31% (84 out of 267), *P* = 0.0001, two-tailed Fisher’s exact test) or with those from lineages that did not show evidence of recall (49% (43 out of 87) versus 19% (16 out of 83), *P* < 0.0001, two-tailed Fisher’s exact test). However, recall was significant only after controlling for both factors (adjusted odds ratio = 3.911, 95% confidence interval (CI): 1.983–8.002; Supplementary Table 3).

**Extended Data Fig. 3:**
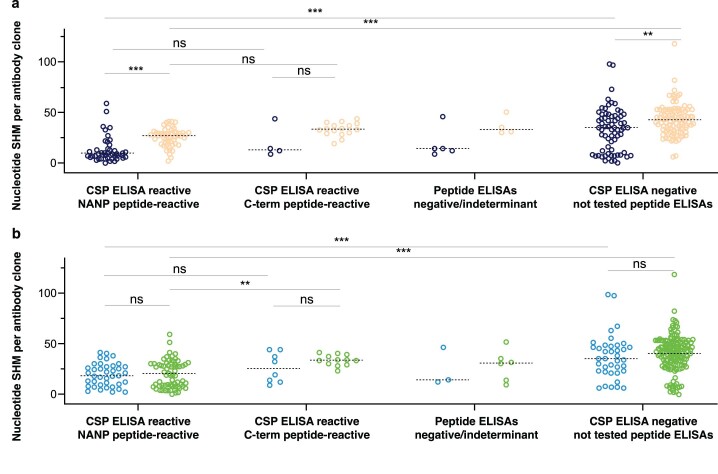
SHM and CSP-peptide binding of mAbs versus RTS,S dose group and vaccinee protection status. **a**, **b**, Number of nucleotide mutations from germline (SHM) for mAbs from expanded lineages of vaccinees, **a**, administered a third standard dose (012 M, dark blue, *n* = 15) or a delayed fractional dose (Fx017M, orange, *n* = 30) of RTS,S with CSP-reactive mAbs shown that are reactive to the repeat region peptide (NANP6, *n* = 45 and *n* = 53, respectively), to the C-terminal region peptide (C-terminal, *n* = 4 and *n* = 16, respectively), or to neither peptide (negative, *n* = 5 and *n* = 4, respectively) in comparison to mAbs not reactive in the CSP ELISA (*n* = 72 and *n* = 113, respectively), and, **b**, from vaccinees that were protected (green, *n* = 36) or unprotected (light blue, *n* = 9) with mAbs shown that are reactive to repeat region peptide (*n* = 59 and *n* = 39, respectively)], to the C-terminal region peptide (*n* = 12 and *n* = 8, respectively), or to neither peptide (negative, *n* = 6 and *n* = 3, respectively) in comparison to mAbs not reactive in the CSP ELISA (*n* = 147 and *n* = 38, respectively), lines are medians, ***P < 0.0001, **P < 0.001, *P < 0.02, or *P* > 0.1 (‘ns’), unpaired two-tailed Mann–Whitney test.

Similar to other reports describing immunization with whole sporozoites^27,32^, we found that many CSP-reactive mAbs had significantly lower levels of SHM than CSP nonreactive mAbs (*P* < 0.0001; Fig. 1d), even after controlling for potential confounders (adjusted coefficient = −14.22, 95% CI: −17.68 to −10.75; Supplementary Table 3), and SHM levels of NANP6-reactive mAbs were lower than those of C-terminal-binding mAbs (*P* < 0.006; Fig. 1d). We also observed that SHM levels of NANP6-binding mAbs were correlated with dose schedule (Extended Data Fig. 3a) but not with P3D protection status (*P* > 0.6; Extended Data Fig. 3b) as previously reported for all IgG sequences^24^.

Surprisingly, the percentages of CSP-reactive (*P* < 0.0007; Fig. 1e) and NANP6-reactive, PB-derived mAbs (*P* < 0.006; Fig. 1e) were lower among P3D-protected vaccinees than P3D-unprotected vaccinees, even after controlling for dose group and convergence (adjusted odds ratio CSP = 0.4109, 95% CI: 0.2512–0.6681; adjusted odds ratio NANP6 = 0.4641, 95% CI 0.2745–0.7845; Supplementary Table 3). This inverse relationship between antigen binding and protection status was also observed when analysis was restricted to only mAbs from the most dominant lineages (rank-size one to four, *P* < 0.0004; Fig. 1e) or when it was extended to include the 20 mAbs from lineages with only one PB (*P* < 0.0005, Fisher’s exact test and *P* = 0.001 by bootstrap analysis; Fig. 1f). These data suggest that simply having higher quantities of circulating PBs expressing CSP- or NANP-reactive antibodies may be insufficient for protection from CHMI.

### Sporozoite-inhibitory antibodies in P3D PBs are not sufficient for P3D protection

**Fig. 2:**
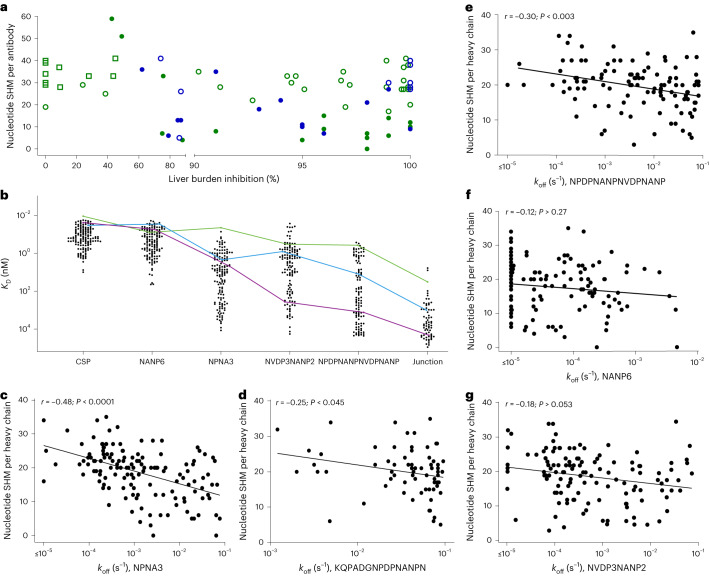
Functional mAbs bind CSP-derived peptides not present in RTS,S. **a**, Percentage inhibition in the sporozoite liver burden mouse model and number of nucleotide mutations from germline are shown for mAbs reactive to either NANP6 repeat-region peptide (circles, *n* = 67) and C-terminal-region peptide (squares, *n* = 10), and are indicated as originating from vaccinees who were either protected (green, *n* = 54) or unprotected (blue, *n* = 23) and who received either the standard (012M, closed symbols, *n* = 30) or fractional (Fx017M, open symbols, *n* = 47) dose. **b**, SPR-determined binding potencies (*K*_d_) of mAbs (*n* = 141) selected from 35 of the most efficacious lineages tested against CSP and a panel of CSP-derived peptides that are either homologous (NANP6, NPNA3) or heterologous (NVDP3NANP2, NPDPNANPNVDPNANP, junction [KQPADGNPDPNANPN]) to RTS,S. Examples are shown of a mAb with a broadly promiscuous binding profile (green, AB-007163), another with a profile relatively biased toward homologous peptides (purple, AB-007143) and a third with a profile between these extremes (blue, AB-007175). **c**–**g**, Simple two-tailed linear regression comparing the number of nucleotide mutations from germline (SHM) per heavy chain versus log-transformed SPR binding off-rate (*k*_off_) against peptides. **c**, Short, major repeat (NPNA3, *n* = 140). **d**, Junctional (KQPADGNPDPNANPN, *n* = 68). **e**, Short, minor repeat (NPDPNANPNVDPNANP, *n* = 109). **f**, Long, major repeat (NANP6, *n* = 141). **g**, Long, minor repeat (NVDP3NANP2, *n* = 129). See Extended Data Table 1 for correlation analyses with nontransformed data. Dissociation rate (*k*_off_) measurements were limited to a minimum of 10^−5^ s^−1^. MAbs, with rates ≤10^−5^ s^−1^ included in the graphic but excluded from correlation analyses.

Given the well-reported protective activity of CSP-binding mAbs in both humans^11,13^ and mice^17,20,25,26,33,34^ and the surprising inverse association between CSP reactivity and vaccinee protection status, we selected 77 mAbs (Methods) from both protected and unprotected vaccinees for advancement in vivo^21,35^. Over half of these mAbs (44 out of 77) provided ≥95% inhibition of sporozoite liver burden, with some demonstrating near-complete protection (≥99.9% inhibition) in C57BL/6 mice (*n* = 10) infected intravenously with chimeric *Plasmodium berghei* (Pb) sporozoites expressing PfCSP rather than PbCSP. All 44 mAbs bound the NANP-repeat region of CSP, with most being derived from the IGHV3-33 germline. Thirteen other NANP-binding, IGHV3-30/33 mAbs demonstrated limited inhibition of parasite liver burden (80–95%) and 12 mAbs, including three C-terminal peptide binders, showed minimal inhibition (20–80%; Fig. 2a and Supplementary Table 2).

Roughly one-third of mAbs tested in vivo (30%, 23 out of 77) were from unprotected vaccinees, including half (7 out of 14) that showed near-complete protection in mice (≥99.9% inhibition; Fig. 2a and Supplementary Table 2), but neither P3D protection status nor vaccinee dose regimen remained significantly correlated with mouse liver burden inhibition in a multivariable linear regression model (Supplementary Table 3). These data suggest that PB expression of these inhibitory antibodies alone is insufficient to drive protection against CHMI. For example, the highly effective antibody AB-000317 (ref. ^20^) was observed in both a protected and an unprotected vaccinee (Fig. 1f, red circles). However, the AB-000317 lineage was the largest PB lineage in the protected vaccinee but the seventh rank-size lineage in the unprotected vaccinee (respectively, 15.8 versus 1.7% of PB in expanded P3D lineages,). While anecdotal, these data are consistent with the hypothesis that antibody-mediated protection induced following RTS,S is driven by a confluence of immunological factors including, but not limited to, B cell receptor recombination and affinity maturation, as well as other factors not measured here including anti-CSP titer in blood, the contribution of other B cell and T cell subsets and/or other immune factors^36^.

### Inhibitory antibodies from vaccinees bind CSP peptides not included in RTS,S

To explore the developability of these inhibitory mAbs as potential drugs, 35 NANP-repeat-binding lineages originating from CHMI-protected vaccinees were selected for further pharmacology studies from the 52 that demonstrated ≥90% inhibition in the sporozoite-challenge screen (Methods). To avoid sequence features that can potentially limit drug developability, and to survey lineages with extensive clonal diversity, multiple clones were chosen from 23 of the 35 lineages. Overall, 141 mAbs from 21 protected vaccinees representing both RTS,S dose regimens and a range of SHM levels were selected for testing.

**Extended Data Fig. 4:**
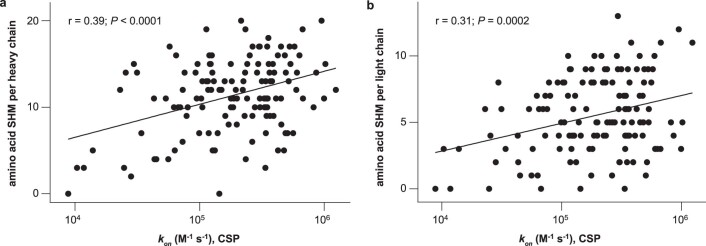
CSP-binding on-rates and amino acid divergence from germline. **a**, **b**, Simple two-tailed linear regression of log-transformed SPR binding on-rates (*k*_*on*_) to CSP of mAbs (*n* = 141) from 35 of the most efficacious lineages compared to the number of, **a**, heavy, and, **b**, light chain amino acid mutations from germline (SHM). For correlations of non-transformed data, see Extended Data Table 1.

These downselected antibodies displayed a broad range of affinities against CSP as measured by surface plasmon resonance (SPR) (CSP-binding affinity, *K*_d_, of 11 pM–9.8 nM; Fig. 2b and Supplementary Table 4) and demonstrated a significant association between *K*_d_ and nucleotide SHM levels (*P* < 0.004, *r* = −0.27 and *P* < 0.0003, *r* = −0.34 for heavy and light chain, respectively, Spearman test), consistent with affinity maturation to CSP that occurred following RTS,S. Correlations between *K*_d_ and heavy- and light-chain SHM levels are probably driven by the binding association rate (*k*_on_) to CSP (*P* < 0.0005, *r* = −0.29, Spearman) because the correlations between dissociation rates (*k*_off_) and SHM levels were not significant (Extended Data Fig. 4a,b and Extended Data Table 1).

Antibodies were also evaluated by SPR for binding to short (12–15 residues) and long (20–24 residues) peptides derived from the varied tetrapeptide-based homologous (NPNA3 and NANP6 peptides) and heterologous (NPDPNANPNVDPNANP, NVDP3NANP2) and junctional [KQPADGNPDPNANPN]) peptides of CSP^16,17,20,26,28,34,37,38^ (Fig. 2b). Among the strongest correlations we observed were inverse relationships between SHM and *k*_off_ to the short major-repeat peptide, the short minor-repeat peptide and the JR peptide (Fig. 2c–e and Extended Data Table 1). *k*_off_ rates calculated against the long homologous and heterologous peptides and against CSP either show weaker, but still statistically significant, correlations with SHM levels or altogether insignificant correlations (Fig. 2f–g and Extended Data Table 1). This was unexpected given that the long version of the homologous peptide and CSP contain more repeats of the same epitopes present in the short peptide. However, it could be that the multiple tetramers in the NPNA6 peptide may allow for certain antibodies to bind via multivalent avidity^20,39–42^, resulting in slow dissociation with *k*_off_ estimates that were limited to 10^−5^ s^−1^, the lowest *k*_off_ to be accurately determined during data analysis. Because these antibodies were excluded from correlation analyses (Fig. 2, Extended Data Table 1 and Methods), we may have underestimated the relationship between SHM levels and avidity to the long NANP peptide. Taken together, these binding data are consistent with reports that some anti-CSP protective mAbs display promiscuous binding across distinct CSP epitopes^16,20,26,28,34,37,43,44^. Thus, we hypothesized that maturation of these inhibitory mAbs to short homologous peptide sequences may also have benefited binding to heterologous peptides.

### Anti-sporozoite activity correlates with CSP-peptide binding and SHM levels

**Fig. 3:**
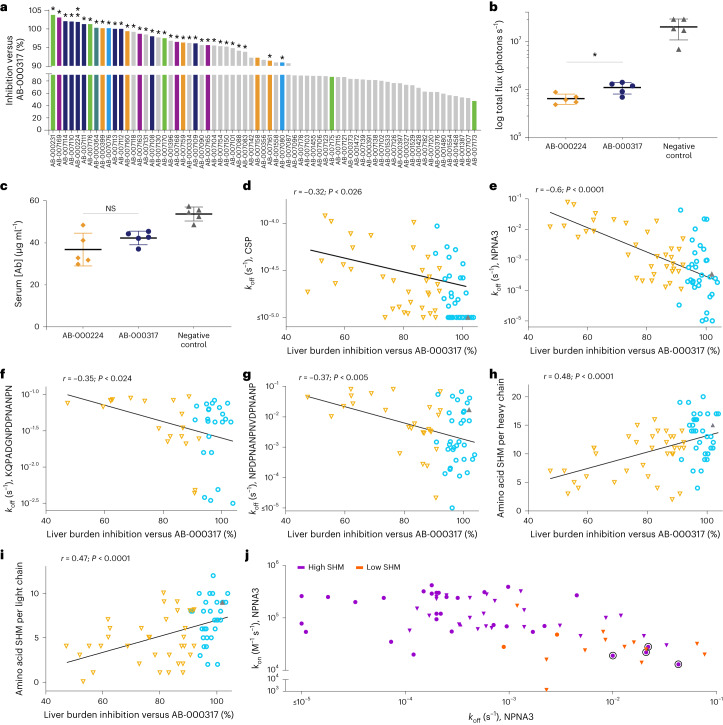
In vitro binding, in vivo activity and SHM associated with developable mAbs. **a**–**c**, Liver burden model. **a**, Percentage inhibition compared with untreated, infected mice (geometric mean, *n* = 5, 100 μg per mouse) normalized to the activity of AB-000317 of *n* = 69 antibodies (32 lineages, *P > 0.05, **P < 0.05 activity greater than AB-000317, no icon *P* < 0.05 activity less than AB-000317, two-tailed, nonparametric log-rank), with colors other than gray indicating the six lineages containing the most efficacious mAbs. **b**,**c**, Example data from AB-000224 and AB-000317 of parasite bioluminescence in the liver (total flux, photons s^−^^1^), *P = 0.03 (**b**) and serum concentrations (serum(Ab), μg ml^−^^1^) of mAb at the time of sporozoite challenge (**c**), *P* > 0.2 (NS), mean ± s.d. (*n* = 5 mice), two-tailed Mann–Whitney test. **d**–**i**, Simple two-tailed linear regression of percentage liver burden inhibitory activity compared with untreated, infected mice (geometric mean, *n* = 5) and normalized to the activity of AB-000317, with each mAb indicated as having activity either significantly better (gray upward-pointing triangle), not different to (cyan circles) or weaker than (orange downward-pointing triangles) AB-000317 (two-tailed, nonparametric log-rank) versus **d**–**g**, log-transformed binding off-rate (*k*_off_) against CSP (*n* = 70) (**d**), major repeat (NPNA3, *n* = 70) (**e**), junctional (KQPADGNPDPNANPN, *n* = 42) (**f**) and short minor-repeat (NPDPNANPNVDPNANP, *n* = 60) peptides (**g**) and versus **h**,**i**, the number of amino acid changes from germline (SHM) for each mAb (*n* = 69)—heavy (**h**) and light chain (**i**) (see Extended Data Table 1 for correlation analyses of non-log-transformed data). Dissociation rate (*k*_off_) measurements were limited to a minimum of 10^−5^ s^−1^ and mAbs were excluded from correlation analyses if assigned this value. **j**, log-transformed SPR binding off- (*k*_off_) versus on-rates (*k*_on_) against NPNA3 peptide of mAbs with either high SHM (purple, ≥20 nucleotide mutations per clone, *n* = 55) or low SHM (orange, <20 nucleotide mutations per clone, *n* = 14) and with activity weaker (downward-pointing triangles), not different to (circles) or better than (upward-pointing triangle) AB-000317 (two-tailed, nonparametric log-rank). mAbs from a lineage reported to bind CSP with Fab–Fab homotypic interactions are indicated (black circles, AB-000399 (refs. ^41,42^), AB-007159, AB-007160, AB-007161).

Seventy mAbs, representing 33 of the 35 protective lineages evaluated in binding studies, were directly compared in a sporozoite-challenge mouse model with the highly efficacious mAb AB-000317 (refs. ^20,21,28,34,38,40,41,45^) to prioritize mAbs for development. Antibodies inhibited 44.1–97.5% of sporozoite liver burden (47.4–103.8% of AB-000317 inhibition; Supplementary Table 4). Overall, about one-half of the mAbs demonstrated inhibition comparable to AB-000317 (*n* = 32) while the other half demonstrated significantly weaker inhibition (*n* = 36). One antibody, AB-000224, demonstrated superior activity to AB-000317 (Fig. 3a,b and Supplementary Table 4). Serum concentrations in mice for most mAbs were at least 1,000-fold higher than the CSP *K*_d_ of the respective mAbs (Supplementary Table 4 and Fig. 3c), indicating that antibodies demonstrating weak inhibition were not likely to be due to low levels of circulating antibody. Lineages with at least one mAb that demonstrated activity consistent with AB-000317 were considered for further advancement.

To determine whether RTS,S-driven affinity maturation may have contributed to in vivo activity in this second set of downselected mAbs, we assessed whether peptide-binding kinetics or SHM levels correlated with percentage inhibition in the liver burden model. Inhibitory activity was associated with slower *k*_off_ rates from CSP (Fig. 3d), from the short homologous peptide, (Fig. 3e), from the heterologous JR (Fig. 3f) and from the short (Fig. 3g) and long minor-repeat (*P* < 0.002, Spearman; *P* > 0.3, Pearson) peptides (Extended Data Table 1). Consistent with the hypothesis that maturation to short NANP sequences may induce more inhibitory activity than multiple repeats of NPNA-based epitopes^37,46^, we did not observe significant correlations between inhibitory activity and binding kinetics with the long homologous peptide NANP6 (*P* > 0.3 (*k*_off_); *P* > 0.7 (*k*_on_), Spearman and Pearson, respectively; Extended Data Table 1). Further, the total number of nucleotide and amino acid changes from germline are significantly correlated to percentage inhibition in the liver burden model, where mutational burden was treated as either a continuous (Fig. 3h,i and Extended Data Table 1) or a categorical variable. In the latter model, antibodies with lower mutational burden (86% (12 out of 14) mAbs with <20 nucleotide mutations) demonstrated significantly weaker inhibition compared with AB-000317 than more mutated mAbs (44% (24 out of 55) mAbs with ≥20 mutations, *P* = 0.007; Fisher’s exact test, two-tailed). Taken together, these correlations suggest that maturation to NANP-based epitopes in RTS,S may have a bystander affect that enhances binding to structurally similar but heterologous sequences and may be functionally important^16,20,26^.

Despite the correlations among SHM levels, binding kinetics and inhibitory activity, some mAbs with high SHM levels are exceptions. In some cases, high-SHM mAbs have poor binding kinetics (slower than median *k*_on_ values and faster than median *k*_off_ values in comparison with the short homologous peptide) and are poor inhibitors compared with AB-000317 (Fig. 3j; 23% (16 out of 69)). These antibodies may have resulted from inefficient affinity maturation and/or aberrant selection mechanisms related to survival in and/or recall from memory^32,37,47–49^ (Fig. 1e–f). In other cases, some high-SHM mAbs have similarly unfavorable binding kinetics but still demonstrate strong inhibitory activity (Fig. 3j; 12% (8 out of 69)). In these latter cases, affinity maturation toward antibody homotypic Fab–Fab interactions may contribute to anti-CSP-binding potency and increased functional activity^27,39,50^, and have been reported for some of the mAbs described here^39,41,42^. Such homotypic interactions may not be reflected in binding kinetics to the short NPNA3 peptide which, due to its short length, cannot sterically accommodate multiple simultaneous binding events^27,39,42^.

Indeed, four of the eight mAbs that have unfavorable binding kinetics but are comparable to AB-000317 in activity originate from a lineage containing a mAb that binds via Fab–Fab homotypic interactions (AB-000399 (refs. ^41,42^); Fig. 3j, red circles). Overall, these data are consistent with mAb affinity maturation via multiple different modes of binding^20,39–42^ and reveal >30 mAbs with activity comparable to that of AB-000317 and the potential for development to clinical leads.

### Lead antibodies prioritized for development

**Fig. 4:**
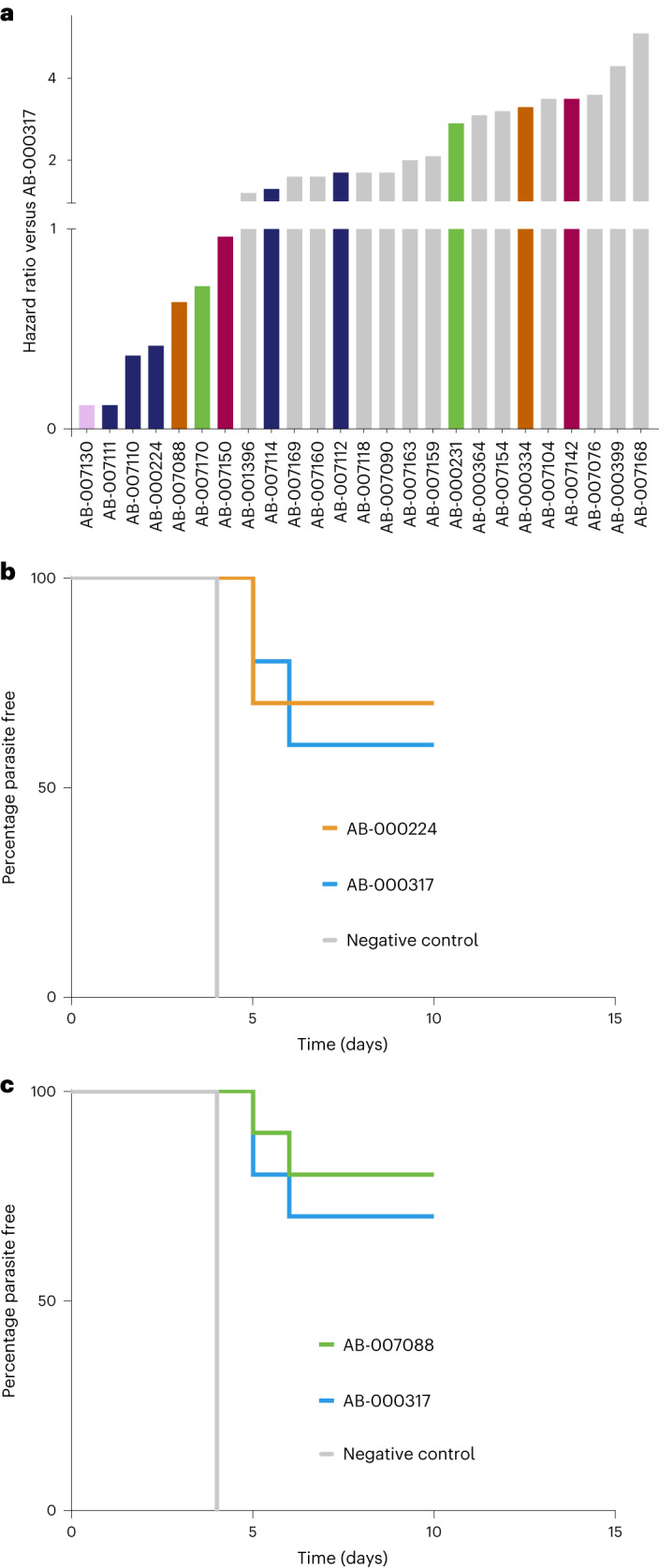
Inhibition of mosquito-bite parasitemia from mAbs prioritized for development. **a**, Hazard ratios of *n* = 25 antibodies (14 lineages) compared with AB-000317 in the mosquito-bite parasitemia model (*n* = 10 mice, 150 μg per mouse), with colors other than gray indicating the five lineages containing the most efficacious antibodies. **b**,**c**, Survival curves from repeat experiments in comparison with AB-000317 and AB-000224 (0.74 (0.15, 3.8)) (**b**) and AB-007088 (0.61 (0.097, 3.8)) (**c**); *n* = 10 mice, two-tailed, nonparametric log-rank (Mantel–Haenszel hazard ratio (95% CIs)).

To identify the most optimal lineage(s) for clinical development, we compared mAbs in regard to the pharmacological and biophysical characteristics necessary for successful manufacture and formulation^9^. Using the sporozoite liver burden data, we further downselected to 26 mAbs representing 15 lineages for evaluation in the parasitemia challenge model, in which mosquitoes infected with chimeric Pb parasites encoding full-length PfCSP fed on C57Bl/6 mice (*n* = 10). Blood-stage malaria infection was determined by blood smear on days 4–10 (refs. ^21,35^). This set of mAbs included AB-000317, AB-000224, 23 other mAbs with liver burden inhibitory activity similar to AB-000317 and one mAb with activity weaker than AB-000317. All except two mAbs were significantly more likely to prevent parasitemia than the negative control. Seven mAbs, including AB-000224 and two other mAbs from the same lineage, displayed a trend towards superior protection versus AB-000317 (nonparametric log-rank hazard ratios <1 versus AB-000317; Fig. 4a and Supplementary Table 4). Serum concentrations in mice for almost all mAbs (25 out of 26) at the time of infection were at least 1,000-fold higher than the respective *K*_d_ CSP–SPR of the mAbs (Supplementary Table 4), indicating that mAbs more efficacious than AB-000317 were probably not missed due to low levels of circulating antibody. Overall, the data suggest that AB-000317 and AB-000224 have in vivo activity at or near maximal efficacy among this set of lead antibodies. Given the previous developability concerns with AB-000317, we selected AB-000224 as the lead molecule.

**Fig. 5:**
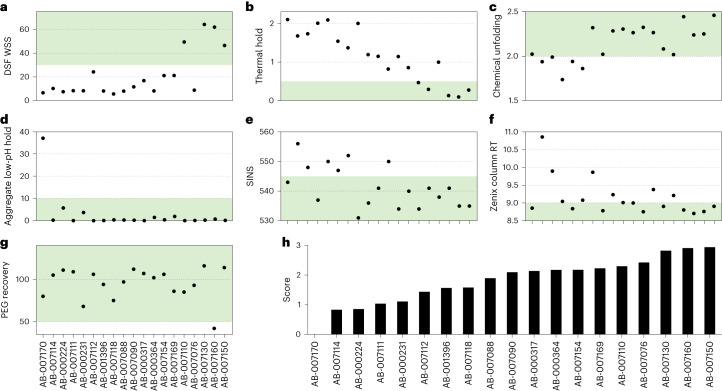
Developability properties of lead antibodies. **a**–**g**, Developability properties of prioritized lead antibodies as characterized by scores for DSF WSS (**a**), thermal hold (**b**), chemically induced unfolding (**c**), low-pH stability (aggregate low-pH hold) (**d**), SINS (**e**), stand-up monolayer affinity chromatography (Zenix column RT) (**f**) and relative solubility analysis (PEG recovery) (**g**); green shading indicates preferred ranges. **h**, Relative aggregate scores generated from assay panel results.

While functional potency is essential for any effective drug, biophysical properties including manufacturability, stability and formulation are equally critical for successful drug development^51,52^. Thus we assessed selected drug properties (for example, protein conformational and solution colloidal stability; Methods) for AB-000224 and 18 other mAbs representing 14 lineages with in vivo activity comparable to that of AB-000317 (Fig. 5). Although none of the data indicate that any of the leads should be eliminated due to development risks, the prime lead (AB-000224) and its siblings demonstrated less favorable characteristics than other mAbs, including relatively poor biophysical stability and aggregation properties (Fig. 5). Thus we selected AB-007088, which demonstrated a similar trend in superiority over AB-000317 in a repeated parasitemia challenge experiment (Fig. 4c and Supplementary Table 4), as the backup molecule given its more favorable developability characteristics (Fig. 5).

### Clinical candidate engineered for optimized developability

**Fig. 6:**
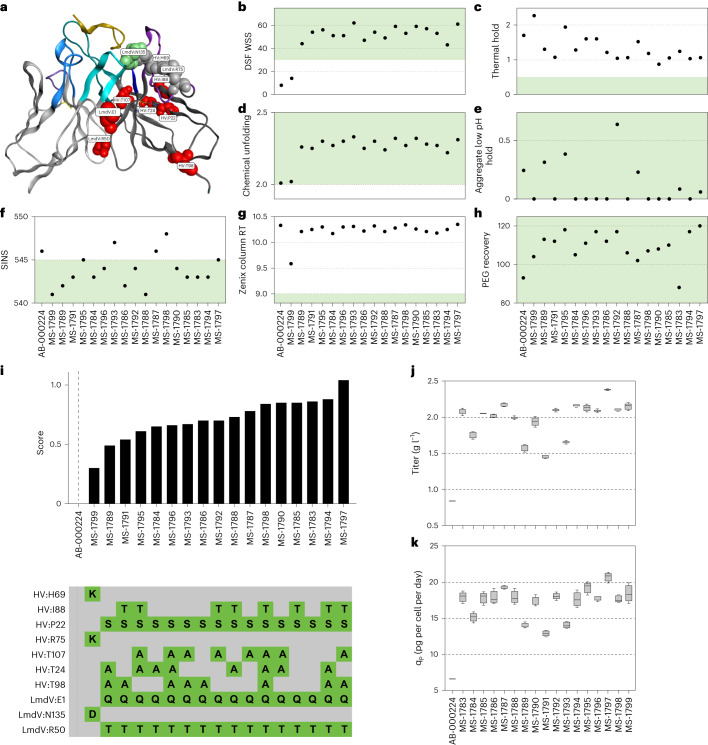
Developability properties of engineered variants from selected lead AB-000224. **a**, Mutations made to generate engineered variants depicted in the Fv region of the Fab of AB-000224 in complex with NPNA4 (gold ribbon, PDB ID 6WFY; Methods). The light-chain framework is shown in silver and the heavy-chain in pale gray. The CDRs for light and heavy chains are indicated, respectively, as CDR1 (light and dark blue), CDR2 (light and dark purple) and CDR3 (light and dark cyan). Mutation sites of engineered variants are labeled as stability violations (red), deamidation sites (green) and paired sibling sites (gray; Methods). **b**–**h**, Developability properties of engineered variants of AB-000224 as characterized by scores from DSF WSS (**b**), thermal hold (**c**), chemically induced unfolding (**d**), low-pH stability (aggregate low-pH hold) (**e**), SINS (**f**), stand-up monolayer affinity chromatography (Zenix column RT) (**g**) and relative solubility analysis (PEG recovery) (**h**); green shading tindicates preferred ranges. **i**, Aggregate score of assay panel results from **b**–**h** for each engineered variant, with mutations shown in comparison with parental AB-000224. **j**,**k**, Characterization of stable cell production pools generated from engineered variants compared with AB-000224 for production titers (g l^−^^1^) (**j**) and cell-specific productivity (*q*_P_, pg per cell per day) (**k**). Boxes indicate interquartile ranges, lines within boxes are medians and whiskers represent minimum and maximum (four independent replicates of each antibody).

**Extended Data Fig. 5:**
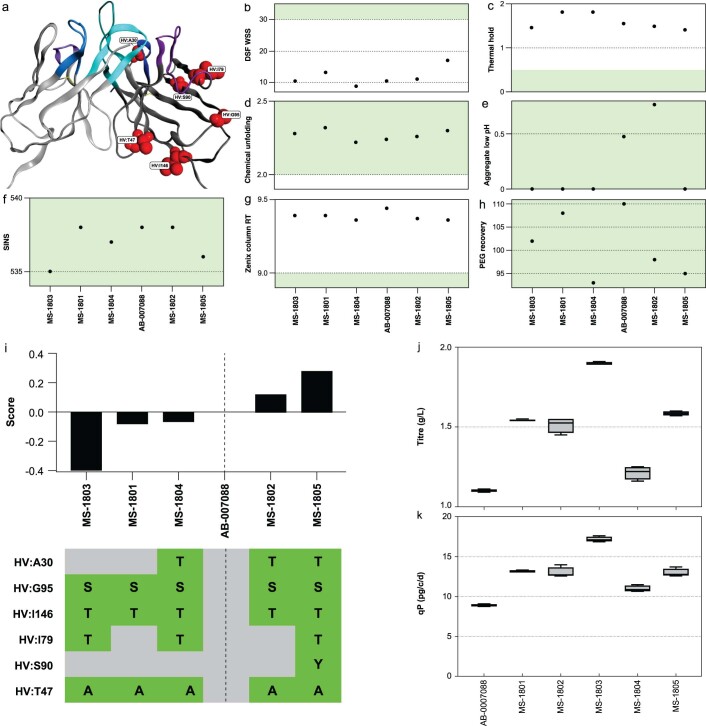
Generation and developability properties of engineered variants from backup molecule. **a**, Mutations made to generate engineered variants are depicted in the Fv region of a Fab structure model of AB-007088 built in the MOE Antibody Modeler (*see* Methods). The light chain framework is in silver and the heavy chain in dim grey. The CDRs for light and heavy chains are indicated, respectively, as CDR1 (light and dark blue), CDR2 (light and dark purple), and CR3 (light and dark cyan). Mutation sites of engineered variants are labelled as stability violations (red, *see* Methods). **b–h**, Developability properties of engineered variants of parental backup, AB-007088, as characterized by scores from, **b**, DSF WSS, **c**, thermal hold, **d**, chemically induced unfolding, **e**, low pH stability (aggregate low pH hold), **f**, self-interaction nanoparticle spectroscopy (SINS), **g**, stand-up monolayer affinity chromatography (Zenix column RT), **h**, relative solubility analysis (PEG recovery) with green shading that indicates preferred ranges. **i**, Aggregate score of assay panel results from **b-h** for each engineered variant with mutations shown in comparison to AB-007088. **j–k**, Characterization of stable cell production pools generated from engineered variants compared to AB-007088 for, **j**, production titres, (Titre, g/L) and, **k**, cell-specific productivity (qP, pg per cell per day). Boxes indicate interquartile ranges, lines within boxes are medians, and whiskers represent minimum and maximum (4 independent replicates of each antibody).

**Extended Data Fig. 6:**
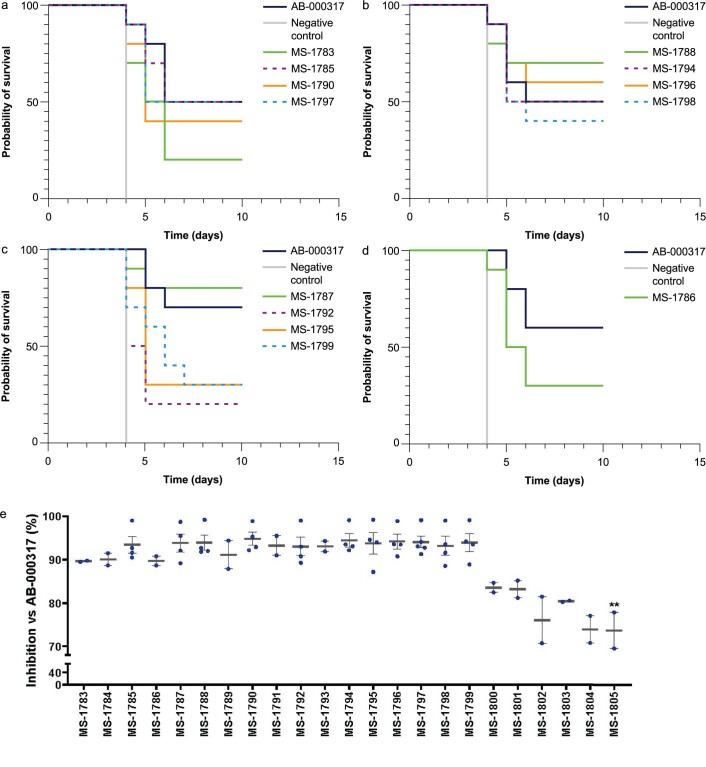
In vivo activity of engineered mAb variants. **a–d**, Mosquito-bite parasitaemia survival curves for 13 variants (*n* = 10 mice), including the eight that had the best aggregate score from the developability assays. **e**, Sporozoite liver burden percent inhibition compared to untreated, infected mice (geometric mean, *n* = 5) and normalized to the activity of AB-000317, lines and bars indicate mean ± SEM, 2–5 independent experiments per mAb, **P = 0.005 or *P* > 0.05 for all other variants, two-tailed Dunn’s multiple comparisons test verses AB-000317 (see Extended Data Table 3 for details).

To address developability risks, we engineered AB-000224 and AB-007088 by mutation of specific residues, primarily in the antibody framework regions, according to the Just—Evotec Biologics’ Abacus design platform to avoid impacting complementarity-determining region (CDR)-mediated binding activity (Fig. 6a,i, Extended Data Fig. 5a,i and Methods). Specifically, outlier amino acid resides in framework regions across germline genes were identified via computational covariance evaluation^53^ of structurally aligned residue positions. These amino acids were replaced with mutations known to impact molecular stability^54^, which often translates to improved developability. For AB-000224 and AB-007088, a total of 17 and five clonal variants, respectively, were designed and tested in the same biophysical and pharmacologic assays used previously. Engineered mutations improved both conformational and colloidal stability of many variants, including enhanced thermal stability (assessed by differential scanning fluorimetry (DSF) and thermal fold assays), solubility and aggregation profiles during storage (assessed in the stand-up monolayer affinity chromatography assay; Fig. 6b–i and Extended Data Fig. 5b–i). Most of the variants retained parental mAb binding profiles against a subset of tested peptides (NPNA3 and NVDP3NANP2; Extended Data Table 2), and activity was not significantly different from AB-000317 for the subset tested in vivo. Unlike the previous screening result that showed AB-000224 to be more efficacious than AB-000317 in the liver burden model (Fig. 3b), average percentage inhibition for AB-000224 and most AB-000224 variants was comparable to AB-000317 (Extended Data Fig. 6 and Extended Data Table 3) despite lower sera antibody levels in some cases (Extended Data Table 3).

Variants were generated as human IgG1 with an Fc mutation that extends antibody half-life (Xtend^55^) and which should compensate for the lower serum antibody levels observed in the mouse studies. These variants were used to make a panel of stable transfectant cells, and antibody production data were collected (Fig. 6j,k and Extended Data Fig. 5j,k) to identify optimal pools for the generation of a production cell line (CHO-K1 derived; Methods). Optimization of stability violations in AB-000224 greatly improved production titers (Fig. 6j,k) which, importantly, can reduce cost per dose in alignment with WHO guidelines^9^. Three cell lines from the panel of 22 variants were selected for clone generation. The best-producing clonal cell line identified in continuous-perfusion bioreactors was from engineered mAb MS-1797 (Fig. 6j,k). Based on this outcome and its superior scores in developability assays (Fig. 6b–i), MS-1797, renamed MAM01, advanced into production following good manufacturing practices. Material generated from this line is being used to support studies for clinical development (NCT05891236) of an antimalaria drug suitable for use in pediatric populations living in LMICs.

## Discussion

Using single-cell sequencing of B cells from RTS,S vaccinees, we generated a library of CSP-specific mAbs that could be assessed and downselected to those most amenable for engineering and development as antimalaria medicines. In doing so we also uncovered important characteristics of the humoral response to RTS,S that may underlie the efficacy and durability of this vaccine.

First, we unexpectedly discovered an inverse relationship between the percentage of CSP-specific, IgG-expressing PBs and vaccinee protection status P3D (Fig. 1e–f). These data suggest that, despite the well-reported association between anti-CSP antibodies and protection following RTS,S^56–60^, more cells expressing anti-CSP or anti-NANP antibodies may not drive stronger protection. Because antibodies targeting repeat regions demonstrate a range of sporozoite-inhibitory activity in vivo, the difference between protective and unprotective antibody repertoires in humans could be driven by the relative proportion of highly versus weakly effective repeat-binding antibodies. This point is exemplified by the highly efficacious mAb, AB-000317, which was expressed in the most dominant P3D lineage of a protected vaccinee and in a much less frequent lineage of an unprotected vaccinee (Fig. 1f). Competition between antibodies at the sporozoite surface (‘epitope masking’)^45,47,48^ and/or within lymphoid organs^32^, where dominant but weakly functional antibodies outcompete subdominant, highly effective antibodies for binding to the repeat regions, could be one explanation for this inverse relationship between protection and prevalence of repeat-binding lineages (Fig. 1e). Indeed, our data indicating that P3D PB repeat-binding mAbs have lower levels of SHM than other mAbs (Fig. 1d) support the hypothesis that immature clones are preferentially activated and expanded over more protective memory clones^32,48,49^. Furthermore, this hypothesis could underlie other observations about RTS,S responses: specifically, functional antibodies in sera were higher post second dose (P2D) versus P3D^56^ and anti-CSP P2D, but not P3D, PB and memory B cells associate with P3D protection status^61^ and some vaccinees lost previous protective immune signatures P3D^61^. Overall, we propose that expression of potent, inhibitory antibodies by P3D PBs alone is insufficient for protection. Rather, relative levels of effective versus ineffective repeat-binding antibodies may be important in provision of consistent protection. Together, these data highlight the need for studies to correlate protection with the ratio of effective to ineffective repeat-binding antibodies circulating in sera at the time of infection.

Second, we found that sporozoite-inhibitory activity in mice does not correlate with binding kinetics to the long NANP6 peptide (Extended Data Table 1) but does significantly correlate with *k*_off_ to CSP and with binding kinetics to both the short NANP-containing peptide (NPNA3) and peptides from the minor-repeat region and JR (Fig. 3d–g and Extended Data Table 1). These data suggest that protective antibodies induced following RTS,S vaccination probably affinity mature to short NANP repeats. Because the short PNANPN sequence is contained within both major repeats (included in RTS,S) and the minor repeat and JRs (not included in RTS,S), affinity maturation against this sequence may allow these antibodies to gain and/or improve promiscuous binding activity to epitopes containing PNANPN or a portion thereof, that may be important for potency^28,39,44^ but which are not fully represented in RTS,S. Consistent with this interpretation, aggregate levels of SHM in both heavy and light chains of inhibitory mAbs are correlated with binding kinetics to NANP-, NVDP- and NPDP-containing short peptides (Fig. 2c–e and Extended Data Table 1), as well as with inhibitory activity in the sporozoite-challenge model (Fig. 3h–i and Extended Data Table 1). Overall, the data are consistent with suggestions that next-generation anti-CSP vaccines contain fewer NANP repeats^37,62^ and/or should include sequences from minor repeats and JRs^16,28,34,46,63–66^.

Last, multiple observations including (1) the inverse relationship between the percentage of CSP- and NANP6-binding antibodies from expanded linages and protection against CHMI (Fig. 1e); (2) the correlation between binding kinetics with NVDP- and NPDP-containing peptides absent in RTS,S (Fig. 3f–g and Extended Data Table 1) and sporozoite inhibition in vivo; and (3) the correlation between binding kinetics to the short NPNA3, but not the longer NANP6 peptide, and sporozoite inhibition in vivo (Fig. 3e and Extended Data Table 1), are consistent with a hypothesis where multiple NANP repeats act as an immune ‘decoy’^37,67^ or ‘smokescreen’ ^66,68^ that dilutes protective immunity^69–73^. Under this hypothesis, antibody lineages that bind only to homologous epitopes are preferentially expanded over promiscuous mAbs that bind well to both homologous and heterologous epitopes. Because many of the antibodies that bind only to NANP repeats offer limited protection in vivo (Extended Data Table 1), enrichment for these antibodies dilutes the protective capacity of the broader anti-CSP repertoire^32,37,48^. In contrast, promiscuously binding antibodies that can simultaneously bind to multiple^39,42,50^ NANP repeats and heterologous epitopes may drive superior protection in vivo because they can saturate binding sites and further stabilize^39,42,50,74,75^ mAb interactions with both homologous and heterologous epitopes. Consistent with this idea, three of the most active in vivo mAbs, CIS43, L9 and AB-000317, can bind CSP with high stoichimetries^16,20,39^ via two binding events^16^. Further studies that examine the mechanisms of mAb binding to junctional, minor- and major-repeat regions and the interaction with sporozoite inhibition are needed to test this hypothesis.

Our study has a number of limitations. First, we did not assess whether the antibodies we characterized from expanded PB lineages collected 1 week before infection accurately reflected the composition of sera antibodies at the time of infection. Second, the current, WHO-recommended RTS,S vaccine includes the adjuvant AS01E^76^. Our study, however, examined antibodies derived from vaccinees who received RTS,S AS01B^24^. While the two adjuvants contain the same components, they are included at different levels^77^. More work will be needed to assess whether RTS,S/AS01E as used in the field produces antibody repertoires like those characterized here. Third, the correlations we observed between binding off-rates and function (Fig. 3d–i and Extended Data Table 1) were limited to mAbs from protected vaccinees. Further studies will be needed to assess whether similar correlations exist for inhibitory, repeat-binding antibodies derived from unprotected vaccinees. Finally, we tested only a small fraction of the sequences we generated. While we focused our discovery campaign on the larger PB lineages from each vaccinee, 3,334 smaller but expanded lineages remain uncharacterized.

By sequencing PBs, which represent the breadth of Ig sequence diversity that originates from lymphoid reactions following RTS,S vaccination, we deconstructed a part of the humoral response from protected and unprotected vaccinees. We identified lineages with highly protective antibodies in mouse models, screened sequence-diverse clones within those lineages for development-related properties and further engineered a clone to optimize its developability characteristics. These properties will increase the likelihood that regimens can be successfully developed for pediatric populations, which require small-volume, concentrated doses at low viscosity^9^. Given that the in vivo efficacy displayed by MAM01 is comparable to AB-000317, which in turn has activity comparable to or better than that of CIS43 (refs. ^16,34^), we believe that MAM01 will be useful for individuals living in malaria-naïve and -endemic regions and may also meet the WHO’s preferred product profile^9^, including cost-effective dosing for delivery in LMICs. By focusing on properties critical for manufacture and distribution to global pediatric populations^9,23^, in addition to the requirement for functional potency, the work reported here may contribute to prophylactic strategies that aid efforts in the eradication of malaria.

## Methods

All research complied with appropriate regulations, committees and institutional requirements as specifically noted in the relevant subsections below.

### Vaccinees, PB isolation and IgG sequencing

PBMCs were collected as part of a phase 2a clinical trial evaluating the RTS,S/A01 vaccine with fractional third and fourth doses^24^. The protocol was approved by the Walter Reed Army Institute of Research Institutional Review Board and the Western Institutional Review Board, and written informed consent was obtained from each subject before study procedures were initiated (ClinicalTrials.gov identifier: NCT01857869). PBMC samples obtained from trial participants or this study were used exhaustively and are not available.

PB isolation, cloning and sequencing were performed using BD FACSDiva software v.8.0.3 and the previously published protocol^78^ with the following modifications. PBMCs were stained with the following mAbs and dilutions: anti-CD3-FITC (BioLegend, catalog no. 300440, clone UCHT1, 1:100), anti-CD14-FITC (BioLegend, catalog no. 325604, clone HCD14, 1:100), anti-CD19-BV421 (BioLegend, catalog no. 302234, clone HIB19, 1:100), anti-CD20-PE/cy7 (Biolegend, catalog no. 302312, clone 2H7, 1:100), anti-CD27-BV510 (BioLegend, catalog no. 302836, clone O323, 1:50), anti-CD38-A647 (BioLegend, catalog no. 303514, clone HIT2, 1:100), anti-IgA-FITC (Miltenyi, catalog no. 130- 113-475, clone IS11-8E10, 1:50), anti-IgM-FITC (BioLegend, catalog no. 314506, clone MHM-88, 1:50) and anti-IgD-FITC (Biolegend, catalog no. 348206, clone IA6-2, 1:50). IgG^+^ PBs were single-cell sorted into 96-well polymerase chain reaction (PCR) plates containing hypotonic buffer (330 nM dNTPs (NEB, catalog no. N0447L), 1 μg ml^−^^1^ bovine serum albumin (NEB, catalog no. B9000S), 2 mM DTT (Sigma-Aldrich, catalog no. 43816), 0.5% IGEPAL-630 (Sigma-Aldrich, catalog no. I8896) and 500 units ml^−^^1^ Ribolock (Thermo Fisher Scientific, catalog no. EO0384)) based on gating for CD3^−^CD14^−^CD19^+^CD20^−^CD27^+^CD38^+^ + IgA^−^IgM^−^IgD^−^ cells (Supplementary Fig. 9). Sequencing of IgG messenger RNA isolated from single-cell sorted PBs was performed with the following modifications: desthiobiotinylated Oligo(dT) and Maxima H-Reverse Transcriptase (Thermo Fisher Scientific, catalog no. EP0753) were used for reverse transcription (RT), complementary DNA was extracted using Dynabeads MyOne C1 Streptavidin beads (Thermo Fisher Scientific, catalog no. 65001), concentrations of final NGS library preparations were determined using quantitative PCR (qPCR; KAPA SYBR FAST qPCR Kit for Titanium, Kapa Biosystems) and natively paired IgG heavy- and light-chain amplicons were sequenced using Roche GS FLX + 454 Titanium sequencing with system Software v.2.9.

DNA barcode assignment and sequence assembly were performed as previously described^78^ except for the following modification: a minimum coverage of ten reads was required for each heavy- and light-chain assembly. Both heavy- and light-chain reads were required to assemble unique contigs within a well. In cases where there was one or more contig, we rejected that well unless one of the contigs constituted 90% of reads.

### Sequence, lineage and repertoire feature analyses

#### Germline assignments and determination of SHM levels

Variable (V), diversity (D) and joining (J) gene segment assignment and mutation identification were performed using an implementation of somatic diversification analysis^79^ and the IMGT human immunoglobulin germline database release IMGT_202031 (ref. ^80^). Nucleotide SHM substitutions were counted by aligning the heavy and light variable domains (start of framework 1 to end of framework 4) with a hidden Markov model that includes states for germline aligning regions (VDJ for heavy, VJ for light) and N nucleotide regions, and that counts substitutions with respect to the germline sequence in just the aligned portion (not including the rare, observed indels). IgG isotype (IgG1–IgG4) assignment was performed by alignment of sequence 3' of framework 4 to the IMGT human Ig constant-region sequences from IMGT_202031 (ref. ^80^).

#### CDR3 and lineage assignments

Complementarity-determining region 3 (CDR3) sequences were defined by the Kabat annotation plus the first amino acid residue of framework 4, from which CDR3 lengths were calculated. Natively paired IgG sequence clones were assigned to the same lineage if derived from the same vaccinee, had the same IGHV and IGK/LV germline gene assignments, the same heavy- and light-chain CDR3 (H3 and L3, respectively) length and at least 75% nucleic acid sequence identity across concatenated H3 and L3. In some cases, clones with IGHV3-33 and IGHV3-30 (germline genes with high sequence identity) met all the criteria for assignment to the same lineage, except for the difference in IGHV. In these cases clones were assigned to the same lineage. Lineages were assigned a rank-size based on lineage frequency (number of PBs expressing clones in the lineage divided by total number of PBs in the repertoire). Lineages with the same number of PBs were reported to have the same rank-size.

#### Convergence, clonality and recall

Two IgG clones were defined as convergent if derived from different vaccinees, had the same IGHV and IGK/LV germline gene assignments, the same heavy- and light-chain CDR3 (H3 and L3, respectively) length and at least 85% BLOSUM62-weighted amino acid sequence similarity between concatenated H3 and L3. A lineage was defined as convergent with another lineage if they were derived from different vaccinees and if there was at least one IgG clone in the first lineage that was convergent with at least one in the second. Clonality was summarized as normalized entropy across all lineages in each P3D vaccinee repertoire. Specifically, the sum over *i* in 1 to *N* of (*Ki*/*N* × log(*Ki*/*N*))/log(*N*), where *N* equals the number of lineages in the repertoire and *Ki* is the size of each lineage as the number of PBs. Normalized entropy takes values between 0 and 1 inclusive, where 0 implies that a single lineage is totally dominant in abundance and 1 implies that some sets of lineages *N* > 1 are all equally abundant. Recalled lineages were defined as those with at least one PB antibody clone observed in both P3D and P4D repertoires among vaccinees (*n* = 17) from whom at least 100 PBs were sequenced from P4D PBMC samples.

### Selection of lineages and clones for CSP ELISA screening library

Plasmablast lineages (*n* = 369) were selected for initial screening of CSP reactivity as described above (Supplementary Fig. 8a and Supplementary Table 2). A specific clone from each lineage was chosen for recombinant expression and screening based on one or more of the following properties: (1) the clone was the most frequently observed across the PBs of the lineage (‘dominant clone’); (2) the antibody sequence is convergent with at least one other sequence where convergence is described in ‘Convergence, clonality and recall’; (3) the clone was selected from the ‘leafiest descent’ clade of a phylogenetic tree, where each terminal clade is rank ordered by size according to the number of leaves (that is, sequences); and (4) the clone has the greatest number of nucleic acid mutations from germline among all clones in the lineage (‘most mutated clone’). Three clones (0.8% of the screening library) did not meet any of these criteria due to errors that were not detected until after screening had occurred. Supplementary Table 5 shows which criteria apply to each selected clone, with some noted as ‘tie’. In this case, two clones from the same lineage met one of the criteria above and we made a random selection between the two. Proportions of antibodies that meet each criterion from protected and unprotected vaccinees are not statistically different to those seen for all antibodies in the screening library, Fisher’s exact test (Supplementary Table 6).

### Recombinant antibody production

Antibody syntheses were performed by LakePharma, Inc. Each gene sequence was cloned into LakePharma’s proprietary high-expression mammalian vector. Variable regions sequences were synthesized and subcloned into expression vectors containing human heavy-chain IgG1 and appropriate human kappa or lambda light-chain constant-region sequences. Each completed construct was sequence confirmed before plasmid production scale-up. Suspension HEK293 cells (ATCC) were seeded in a shake flask and expanded using serum-free, chemically defined medium. On the day of transfection the expanded cells were seeded into a new flask with fresh medium. Each DNA construct was transiently transfected into HEK293 cells using a cationic lipid transfection method. Cells were maintained as a batch-fed culture until the end of the production run. Conditioned medium from the transient production run was harvested and clarified by centrifugation and filtration. The supernatant was loaded over a Protein A column pre-equilibrated with binding buffer. Washing buffer was passed through the column until the optical density (OD_280_) value (NanoDrop, Thermo Scientific) was measured as zero. The target protein was eluted with a low-pH buffer, fractions were collected and the OD_280_ value of each fraction was recorded. Fractions containing the target protein were pooled and filtered through a 0.2-μm membrane filter. Purified antibodies were dialyzed against PBS and analyzed with LabChip GXII. Endotoxin measurements were performed using the chromogenic limulus amebocyte lysate method with Pyrochrome (Associates of Cape Cod).

### CSP, NANP peptide and C-terminal peptide ELISA

ELISA methodologies have previously been described^24^. mAbs were mapped to CSP using a nearly-full-length CSP, the NANP6 peptide and a CSP C-terminal peptide (Pf16)^81^. All antibodies were evaluated at either 0.15 or 0.04 µg ml^−^^1^ concentrations and classified as ‘positive’, ‘negative’ or ‘indeterminant’. OD values were determined using Biotek Gen5 software v.2. ELISA OD was converted to fold-induction over the average of four negative control antibodies run in each experiment. The range of OD responses observed in each experiment was then used to determine a borderline indeterminant range for that experiment. MAbs were considered negative if the OD was less than the average negative control mAb OD + 3× standard deviations. MAbs with ODs that exceeded the ‘negative’ threshold but were within 20% of the OD range for the experiment were classified as indeterminant. Any antibody OD above the experimental indeterminant threshold was classified as positive.

### HBsAg ELISA

Antibodies and the positive-control PC3 (starting at 150 pM) were evaluated for HBsAg reactivity using a four-point, 1:3 dilution series prepared in duplicate and tested using the MONOLISA Anti-HBs EIA kit (Bio-Rad, catalog no. 25220). The maximal stock input was 10% of the purified total volume for each of the 139 tested antibodies. If the antibody required <10% of the purified total volume the starting concentration was individually adjusted to 300 nM; otherwise the starting concentration was based on the protein amount included in 10% of the purified total volume. The cutoff calibrator from the kit was performed in quadruplicate while both negative controls were each performed in duplicate. Antibodies were considered HBsAg positive if the signal met the cutoff calibrator criteria for at least one concentration ≤30 nM. Antibodies were considered ‘borderline’ HBsAg reactive if the signal was negative at concentrations tested ≤30 nM but did meet the cutoff calibrator criteria for any concentration >30 nM. Antibodies were considered negative if the signal did not meet the cutoff calibrator criteria for any concentration tested.

### Selection of mAbs for initial characterization in the mouse sporozoite-challenge model

Among the 102 mAbs that were reactive in the NANP6 peptide ELISA (Fig. 1d), 69 were selected to be screened in vivo based on IGHV representation, vaccine protection status and SHM levels. These 102 mAbs were divided into 11 groups based on their different IGHV (IGHV1–2, 1–69, 1–8, 3–15, 3–23, 3–30, 3–33, 3–48, 3–49, 3–7 and 5–51). We selected at least half of the mAbs from each IGHV group; these mAbs originated from 26 protected and eight unprotected. vaccinees. Only mAbs with high levels of SHM (≥20 nucleotide mutations from germline per antibody) were selected, except for the sets that contained IGHV3-33 and 3-49, from which some mAbs with low levels of SHM (<20 nucleotide mutations from germline per antibody) were also included. Two mAbs did not express sufficient material for testing in vivo.

Among the 20 mAbs that were reactive in the C-terminal (Pf16) ELISA (Fig. 1d), 11 of the 12 originated from protected vaccinees and were screened in vivo. These mAbs included all of the IGHV germline genes observed among C-terminal (Pf16) binders from protected vaccinees (IGHV3–11, 3–21, 3–30, 3–48 and 4–59). One mAb did not express sufficient material for testing in vivo.

### Selection of mAbs characterized in SPR binding analyses

All of the mAbs that originated from protected vaccinees (*n* = 36) and that demonstrated ≥90% inhibition in the initial sporozoite liver burden mouse model screen were selected for further binding analyses, except for one that was observed to be reactive in the HBsAg ELISA (AB-000239; Supplementary Table 2). For each of these 35 mAbs (representing 35 unique lineages) the original hit mAb was selected if it contained no high-risk liability (that is, odd number of cysteines in CDRs, any canonical N-linked glycosylation sites in CDRs, Fv net charge >9 (at pH 5.5) or hydrophobicity index >6.5). In cases where the original hit had one or more high-risk liabilities, a different clone was selected from the lineage. More than one clone was selected from lineages with extensive interclonal sequence diversity; this was done using the following algorithm: (1) query each clone of lineage in leafiest descent order; (2) skip any clones with more than one high-risk liability; (3) skip any clones overly similar in amino acid sequence to clones already picked and where the distance between clones was determined as the fraction of distinct CDR amino acid positions using the BLOSUM62 matrix (≤0); and (4) adjust the distance that is acceptable between clones so that a total of 141 clones were ultimately selected from the 35 lineages (Supplementary Table 4).

### High-throughput SPR

The binding kinetics measurements of mAb interaction with CSP antigens were made using the Carterra LSA high-throughput SPR platform with Epitope Software v.1.5 and CMD200M sensor chips (Carterra) at 25 °C. The antigen panel included recombinant CSP and synthetic peptides NPNA3 (NPNANPNANPNA) and NANP6 (NANPNANPNANPNANPNANPNANP), junctional peptide (KQPADGNPDPNANPN), NPDPNANP2NVDP (NPDPNANPNVDPNANP) and NVDP3NANP2 (NVDPNANPNVDPNANPNVDP), which were custom made by CPC Scientific. Except for NANP6, all other peptides were acetylated at N termini and amidated at C termini. NANP6 contained an N-terminal biotin-aminohexanoic acid tag and an unmodified C terminus. Two microfluidic modules, a 96-channel print-head (96PH) and a single flow cell (SFC) were used to deliver liquids onto the sensor chip. A single analyte antigen was titrated in each assay against the immobilized antibodies.

Immobilization of antibodies onto CMD200M chips depended on the type of analyte used during titration. In assays involving recombinant CSP used as an analyte, a goat anti-human IgG Fc antibody (Millipore, Sigma-Aldrich AP113, lot no. 3027077) was first immobilized onto the chip through amine coupling. The chip was then activated by 100 mM *N*-hydroxysuccinimide and 400 mM 1-ethyl-3-(3-dimethylaminopropyl) carbodiimide hydrochloride (EDC) (GE Healthcare, mixed 1:1:1 with 0.1 M MES buffer pH 5.5) for 600 s and followed by immobilization of anti-human IgG Fc (in 10 mM sodium acetate pH 4.5) at 50 µg ml^−1^ for 900 s. Unreactive esters were quenched with a 600-s injection of 1 M ethanolamine-HCl pH 8.5. The chip was then exposed to double pulses (30 s per pulse) of 10 mM glycine pH 2.0. CSP-specific mAbs were then captured on anti-Hu IgG Fc surfaces by injection of mAbs at either 10 or 5 µg ml^−^^1^ for 600 s using the 96PH, with 1× HBSTE buffer (10 mM HEPES pH 7.4, 150 mM NaCl, 3 mM EDTA and 0.01% Tween-20) as running buffer and antibody diluent. When CSP-peptide antigens were used as analytes, the chip was activated by *N*-hydroxysuccinimide/EDC for 600 s followed by direct immobilization of CSP-specific mAbs (in 10 mM sodium acetate pH 4.5) injected at either 10 or 5 µg ml^−^^1^ for 600 s using the 96PH. Unreactive esters were quenched with a 600-s injection of 1 M ethanolamine-HCl pH 8.5, then 45 cycles of 1× HBSTE buffer injections with 1× HBSTE as running buffer were used to wash off nonspecifically bound IgG overnight from the sensor chip surface without the use of regeneration buffer. Except for the capture of mAbs by anti-human IgG Fc and washing of nonspecifically bound IgG, the running buffer was 10 mM MES buffer pH 5.5 with 0.01% Tween-20. Unless specified above, the steps were done using the SFC.

During the initial screening each mAb dilution was immobilized onto two separate spots of the same chip, enabling duplicate measurements. The engineered variants were immobilized onto three different spots enabling triplicate measurements.

A twofold dilution series of the antigen was prepared in 1× HBSTE buffer. The top concentration for full-length CSP and all CSP-peptide antigens was 8 µg ml^−^^1^ (0.25 µM for CSP, 2.92 µM for NANP6, 6.41 µM for NPNA3, 3.76 µM for NVDP3NANP2, 4.70 µM for NPDPNANPNVDPNANP and 5.03 µM for N-interface). The antigen at different concentrations was then injected using SFC onto the chip surface, from the lowest to the highest concentration without regeneration, including eight injections of buffer before the lowest nonzero concentration for signal stabilization. For each concentration, data collection involved 120 s of baseline step and 900 s of dissociation steps. The threshold for measurable off-rates was 10^−5^ s^−1^. Due to the sensitivity of the instrument, off-rates slower than 10^−5^ s^−1^ were associated with large standard errors and could not be confidently determined. The duration of the association step was 240 s for full-length CSP and NANP6 antigens and 300 s for all other CSP-peptide antigens. For all assays the running buffer for titration was 1× HBSTE.

The kinetics titration data collected were first preprocessed in NextGenKIT (Carterra) software, including reference subtraction, buffer subtraction and data smoothing. The data were then exported and analyzed using the TitrationAnalysis tool (https://zenodo.org/record/7998652) developed in-house^82^ (https://gatesopenresearch.org/articles/7-107/v1). The specific binding time courses for each antibody construct immobilized on different spots were fitted to a 1:1 Langmuir model to derive *k*_a_ (‘*k*_on_’, association rate constant), *k*_d_ (‘*k*_off_’, dissociation rate constant) and *K*_d_ (dissociation constant) values. The *K*_d_ values determined for antigens with multiple epitope repeats include the avidity effect. The average values of duplicate measurements were reported for each antibody–antigen pair from the initial screening panel. For the engineered variants, average values of triplicate measurements were reported with the following data acceptance criteria: (1) standard error of the estimated *k*_on_, *k*_off_ and *K*_d_ in each replicate ≤20% and (2) fold change for all three parameters within the triplicate ≤3.

### In vivo functional assessments

Mouse studies used 6–8-week-old C57BL/6 female mice (Charles River Labs), maintained at the animal facility of the Johns Hopkins Bloomberg School of Public Health. Mouse housing was maintained at 40–60% relative humidity and a temperature of 68–79 °F), with at least ten room air changes per hour and a 14/10-h light/dark cycle. No animals or data points were excluded from analyses. Experiments were performed in strict accordance with the recommendations in the Guide for the Care and Use of Laboratory Animals of the National Institutes of Health. The protocol was approved by the Animal Care and Use Committee of Johns Hopkins University (protocol nos. MO18H419 and MO21H417).

#### Sporozoite-challenge liver burden mouse models

In the first functional screen (data are shown in Fig. 2a and Supplementary Table 2), mice (*n* = 10) were administered via intravenous tail vein either 100 or 300 µg of mAb (dose specified in Supplementary Table 2) and were challenged 16 h later via intravenous tail vein with 2,000 *P. berghei* transgenic sporozoites expressing the full *P. falciparum* CSP. Forty-two hours later, mice were euthanized and their livers excised to extract RNA to perform qPCR with RT to measure plasmodial 18S ribosomal RNA using forward primer 5′-TGGGAGATTGGTTTTGACGTTTATGT-3′ and reverse primer 5′-AAGCATTAAATAAAGCGAATACATCCTTAC-3′ as previously described^83^. Parasite loads were expressed as *P. berghei* 18S rRNA copy number, and percentage inhibition of load was calculated in comparison with negative controls.

All other liver burden assays were performed as previously described^21^. *Anopheles stephensi* mosquitoes infected with transgenic *P. berghei* sporozoites expressing *P. falciparum* CSP and luciferase were maintained in an incubator at 19 °C. Sporozoites from mosquitoes were harvested at days 20–23 post infection in 2% HBSS-FBS. Mice (*n* = 5) were intravenously (tail vein) administered 100 μg of antibody, unless otherwise indicated, and challenged 16 h later with 2,000 sporozoites injected via the tail vein. Control mice received either irrelevant or no antibodies. Forty-two hours following challenge, mice were injected with 100 µl of d-luciferin (30 mg ml^−^^1^), anesthetized with isoflurane and bioluminescence expressed by liver parasites was measured using an IVIS Spectrum imager with Living Image v.3.2 software (Perkin Elmer). Results are expressed as photons s^−^^1^.

#### Mosquito-bite challenge parasitemia mouse models

The mosquito-bite challenge for evaluation of sterile protection was performed as described by Flores-Garcia et al.^21^. Mice (*n* = 10) were passively immunized with 150 µg of antibody, anesthetized 16 h later and placed for 10 min on the top of cages containing five mosquitoes infected with *P. berghei* sporozoites expressing *P. falciparum* CSP and luciferase. From days 4–10 following challenge, blood smears stained with Giemsa were observed under a light microscope to determine the appearance of parasitemia. Control mice receiving irrelevant or no antibodies were challenged similarly.

### Assessment of mAb concentrations in serum samples

Blood was collected by retro-orbital bleed 15 h following antibody administration (1 h before infection). Capture antibody (AffiniPure mouse anti-human IgG Fc fragment specific, Jackson ImmunoResearch, no. 209-005-098) was adsorbed overnight at 21 °C onto 96-well polystyrene microplates (Immuno Plate Maxisorp, Thermo Fisher Scientific, no. 439454) in PBS (Dulbecco’s PBS, without calcium and magnesium, sterile, pH 7.4; Wisent, no. 311-425-LL) and then washed three times in wash buffer (0.05% Tween 20 (Sigma, no. P2287) in PBS). Microplates were blocked for 1 h at 21 °C with assay buffer (1% bovine serum albumin (Blocker BSA, ThermoFisher, no. 37525) in wash buffer). After washing with wash buffer three times, serum samples from mice and control standards were added in duplicate at serial dilutions in normal mouse serum and then further diluted 100-fold in assay buffer before incubation for 1 h at 21 °C. Control standards consisted of AB-000317 serially diluted at 1.6-fold increments. from 0.146 to 25.6 µg ml^−^^1^. Microplates were then washed three times with wash buffer and incubated with mouse monoclonal anti-human IgG antibody conjugated to horse radish peroxidase-conjugated clone JDC-101 (Southern Biotech, no. 9040-05) in assay buffer for 1 h at 21 °C. Following three washes with wash buffer, peroxidase substrate (TMB, Bio-Rad, no. 1721068) was added followed by a stop solution (650 nm of TMB stop solution, Southern Biotech, no. 0413-01L). Absorbance was measured at 650 nm (Molecular Devices microplate reader using SoftMaxPro GxP v.6.5.1), and concentrations of human IgG in test samples were calculated using the standard curve generated from the control antibody by interpolation of OD values on the five-parameter logistic standard curve (derived from mean ODs of duplicate standard samples) and adjusted according to their corresponding dilution factor. Final sample concentrations were then determined by calculating the average of all concentrations for a sample obtained within the range of the standard curve.

### Developability characterization assays

#### Sample preparation

Samples were first buffer exchanged against ten diavolumes of 20 mM sodium phosphate and 150 mM sodium chloride pH 7.1 (PBS) using a centrifugal filter with a 30-kDa molecular weight cutoff (Amicon), then normalized to 1 mg ml^−^^1^ using a Lunatic protein concentration plate format instrument (Unchained Labs).

#### Biophysical analysis

##### DSF

DSF was used to determine thermal transition temperature(s) and weighted shoulder scores according to a method previously described^54^.

##### Thermal hold

Thermal hold was assessed by placing 30 μl of sample in a PCR plate (Bio-Rad) in replicate wells, one plate column for each sample. The plate was heated using a C1000 thermocycler (Bio-Rad) set to apply gradient heat from 69 to 74 °C for 5 min. Samples were transferred to a clear 384-well plate (Fisher Scientific Company) and analyzed for turbidity by reading absorbance at 350 nm using a Spectrostar Nano plate reader (BMG Labtech).

##### SINS

Self-interaction nanoparticle spectroscopy (SINS) measurements were performed as previously described^84^. Gold nanoparticles (Ted Pella) were conjugated overnight with an 80:20 ratio of anti-human and anti-goat antibodies (Jackson Immuno Research). Unreacted sites were blocked using an aqueous 0.1% (w/v) polysorbate 20 solution. Conjugated gold nanoparticles were concentrated by centrifugation and 95% of the supernatant was removed. Analysis was carried out in PBS (20 mM phosphate, 150 mM NaCl, pH 7.1) at a protein concentration of 0.05 mg ml^−^^1^ reacted with 5 μl of concentrated conjugated gold nanoparticles. After a 2-h incubation, absorbance spectra from 400 to 600 nm were collected using a Spectrostar Nano plate reader (BMG Labtech) at 2-nm resolution. The wavelength maximum of the spectrum peak is reported.

##### Stand-up monolayer absorption chromatography

Stand-up monolayer absorption chromatography measurements were performed as previously described^85^. Retention times were determined using a Dionex UPLC equipped with a Zenix column (Sepax Technologies) and a running buffer comprising 150 mM sodium phosphate pH 7.0.

##### Low-pH stability

Stability during a low-pH hold was determined as previously described^54^. The increase in high molecular weight of the low-pH-exposed sample versus the control sample is reported.

##### Relative solubility

Solubility was assessed as previously described^54^. Analysis was done in PBS buffer (20 mM sodium phosphate and 150 mM sodium chloride pH 7.1) and a final polyethylene glycol (PEG) 10,000 concentration ranging from 7.2% to 10.4%. The remaining soluble protein following PEG incubation is reported.

##### Chemical unfolding

The chemical unfolding assay was completed as previously described^54^, with some modifications. After a 3-day incubation in 32 guanidine hydrochloride concentrations, samples were measured on a Fluorescence Innovations SUPR-UV plate reader (excitation, 275 nm; emission, 300–440 nm). The measured fluorescence intensity at 362 nm was corrected for scattering and stray light, the unfolding curve was generated by graphing each corrected intensity against the guanidine hydrochloride concentration and the inflection point was reported.

### Variant engineering for clinical candidate and backup

The antibody variable domains of AB-000224, AB-007088 and siblings of both antibodies were analyzed using the Just–Evotec Biologics’ Abacus platform. Sequences were placed in a structure-based numbering system (ASN; Supplementary Tables 7 and 8) derived from the AHo^86^ system to add structural positional data to the sequence space and produce consistent alignments across diverse sets of antibody variable-domain sequences. Germline background was evaluated to predict expression problems and evaluate potential solutions for sequence-based liabilities. Signal peptides were evaluated based on germline background to improve expression. Sequence-level evaluation was performed to identify potential N-linked glycosylation sites, nontypical cysteine residues, isomerization and deamidation sites that could impact function, covariance violations (CVVs)^53^ that could impact stability and tryptophan residues in CDR3s that could lead to degradation. CVV sites were evaluated based on severity, sequence location and whether they would be modified across the variant panel or combinatorially. An empirical multiattribute method (MAM)^87^ was run to determine actual posttranslational modification levels to bolster sequence evaluation. The published structure of AB-000224 (6WFY)^41^ and a structural model of AB-007088 (built using the Molecular Operating Environment, 2022.02 Chemical Computing Group ULC) were evaluated for surface properties that could impact developability such as hydrophobic patches leading to aggregation, electropositive patches leading to increased serum clearance rates and electronegative patches leading to increased high-concentration viscosity. These structures were also used to evaluate the possible mutation sites for property optimization to determine their potential impact on CSP binding.

For AB-000224, the IGLV2-8 signal peptide was chosen for the lambda light chain and the IGKV1‑39 signal peptide for the heavy chain. MAM analysis found one low-level deamidation site that appeared to be in contact with CSP based on CSP-binding complex structures; however, given its low-level occurrence and the fact that a stressed sample did not demonstrate increased deamidation, it was removed from consideration in variant designs. The computed CVV sites were found to be strong across both chains, with the majority being in the heavy chain. AB-000224 siblings AB-007110, AB-007111 and AB-007112, which were also active in vivo, were evaluated at the sequence and structure level to determine any potential beneficial modifications to AB-000224. Positions that differed from AB-000224 were considered optimization sites if they were consistent amongst the siblings or of energetic structural interest. This analysis identified three sites, including the deamidation site, and these three positions were mutated and combined in a single variant. Surface patch analysis found nothing of significance. This analysis resulted in 17 combinatorial variant designs, as depicted in Fig. 6i.

For AB-007088, the IGKV1‑39 signal peptide was selected for both the kappa light and heavy chains. MAM analysis found two low-level deamidation sites, although neither appeared structurally able to impact CSP binding given the known CSP-binding complex structures (PDB IDs 5BK0 (ref. ^25^), 6AXL ref. ^20^, 6AZM ref. ^25^, 6BQB ref. ^26^, 6D01 (ref. ^27^), 6D0X ref. ^27^ and 6D11 (ref. ^27^)), and were not included in variant designs. The computed CVV sites were found to be strong only within the heavy chain. Surface patch analysis found some low levels of minor hydrophobic character, which might have increased hydrophobic interactions but, because their repair would probably have degraded function, they were not included in variant designs. This analysis resulted in five combinatorial variant designs, as depicted in Extended Data Fig. 5i.

### Generation and assessment of stable cell lines for production

Sequences were codon optimized, synthesized at ATUM and inserted into transposon expression vector V52 (ref. ^88^). Constant regions consisted of IGHG1*01 and IGLC7*01 for AB-000224 variants, or IGKC*01 for AB-007088 variants. Each variant was transfected into 20 × 10^6 ^CHO-K1 GS KO cells (Horizon Discovery) and maintained in a serum-free, chemically defined CD OptiCHO commercial growth medium (Gibco) with 4 mM l-glutamine. Transposon DNA (18.75 μg) was combined with 6.25 μg of transposase RNA and mixed with EX-CELL 325 PF CHO (Sigma) to a final volume of 50 μl. Cells were electroporated using the long-duration method^89^ in 4-mm gap cuvettes. Cells were electroporated at 200-V voltage, 725-μF resistance and 3,175-Ω capacitance in an Electro Cell Manipulator 630 coupled to a 630B Safety Stand (BTX). Following electroporation, cells were resuspended in 15 ml of fresh growth medium with glutamine and allowed to recover for 2 days in stationary T75 cell culture flasks. Cells were counted on a Vi-CELL XR (Beckman Coulter), seeded for selection in CD OptiCHO medium lacking glutamine at 1 × 10^6^–2 × 10^6 ^cells ml^−1^ and placed in suspension cell culture. Approximately every 2–4 days, pools were monitored for viability and cell density and selective medium were refreshed. Cells were maintained at 36.7 °C in 5% CO_2_ humidified incubators. Shake flasks (Corning, VWR and Thomson) were stored on platforms at a shaking speed of 150 r.p.m. with 50-m orbital diameter while 50-l vented cap tubes (Corning) and 24-deep-well plates (VWR) were stored on platforms at a shaking speed of 220 r.p.m. with 50-mm orbital diameter.

Cells were considered recovered from selection once they demonstrated >90% viability and >1 × 10^6 ^cells ml^−^^1^ viable cell density, and were seeded into a 24-deep-well plate fed-batch production assay. Each variant pool was seeded in quadruplicate at 1 × 10^6 ^cells ml^−^^1^ in 3 ml by centrifugation and resuspended in 80% proprietary BAK004-026 and 20% CD OptiCHO media per well. Cells were counted on days 3, 6, 8 and 10 with Guava easyCyte (MilliporeSigma). Cell counts were analyzed using Guava CytoSoft Data Acquisition and Analysis Software. On days 3, 6 and 8, cells were fed 5% of starting volume with CellBoost 7 A (Hyclone) and 0.5% of starting volume with CellBoost 7B (Hyclone). Glucose levels were measured on feed days and fed to maintain a minimum of 8 g l^−^^1^. On day 10 the supernatants were clarified with 24-well, 0.2-μm filter plates (Thomson) and replicates were pooled, purified by ProteinA resin and buffer exchanged into PBS at 1 mg ml^−^^1^ for further analysis.

### Statistical analyses

*t*-tests, Wilcoxon rank-sum tests, Wilcoxon matched-pairs tests, Kolmogorov–Smirnov tests, analysis of variance, correlation, regression, log-rank survival analysis, univariate and multivariable logistic and linear regressions and Fisher exact tests were performed using GraphPad Prism. Bootstrap analyses were performed using R (cran.r-project.org). Benjamini–Hochberg false discovery rate analysis was performed using the function p.adjust (p.values.list, method = ‘BH’) from R. The number of observations in each analysis is indicated by *n* in both figures and text. Significance is defined as *P* < 0.05. Error bars on figures are standard errors unless otherwise indicated.

### Reporting summary

Further information on research design is available in the Nature Portfolio Reporting Summary linked to this article.

## Online content

Any methods, additional references, Nature Portfolio reporting summaries, source data, extended data, supplementary information, acknowledgements, peer review information; details of author contributions and competing interests; and statements of data and code availability are available at 10.1038/s41591-023-02659-z.

### Supplementary information

Supplementary Information Supplementary Figs. 1–9, including text legend for each.

Reporting Summary

Supplementary Tables 1–8. One Excel file with eight separate worksheets with one Supplementary table on each worksheet.

### Source data

Source Data Extended Data Fig. 1 and Table 1. An Excel file with two worksheets, ‘Data’ and ‘Notes’. Data contains a table with the binding data used to report analyses in Figs. 2b–g and 3d–j, Extended Data Fig. 4 and Extended Data Table 1. Notes describes how replicates of a comparator control were utilized.

## Figures and Tables

**Extended Data Table 1:**
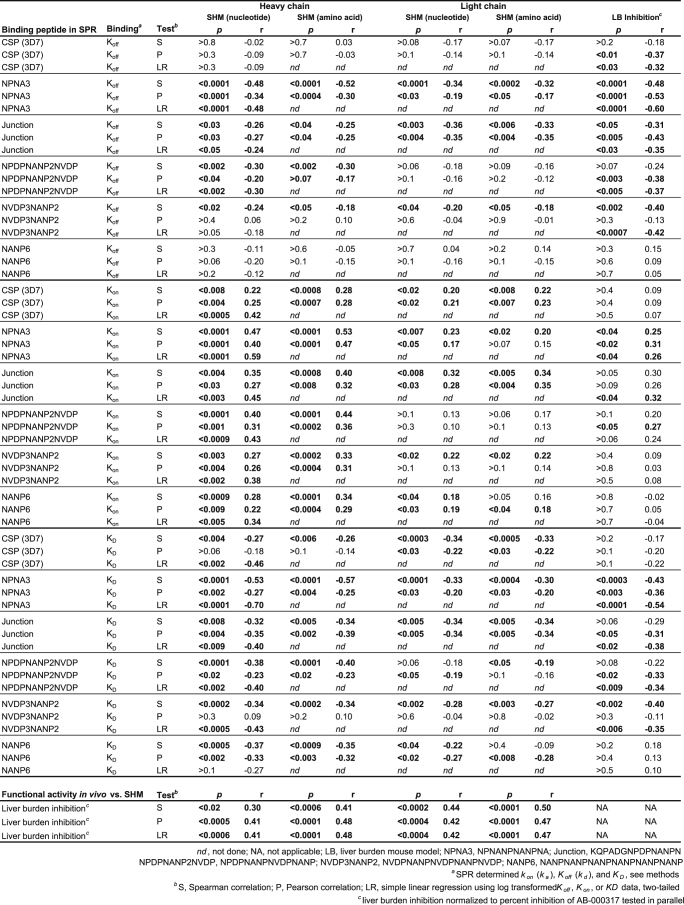
Correlation test results comparing SHM, CSP-peptide binding data, and liver burden inhibition data

Source data

**Extended Data Table 2:**
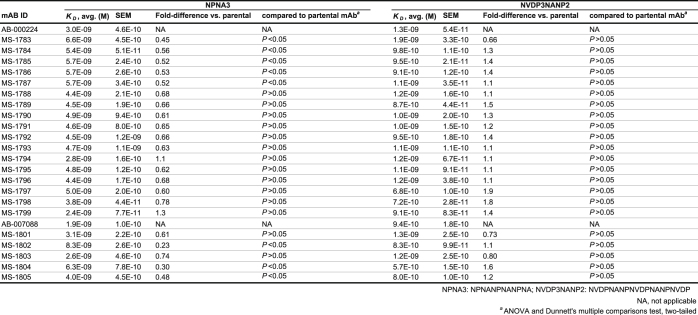
Binding data to CSP-derived peptides of engineered variant mAbs

**Extended Data Table 3:**
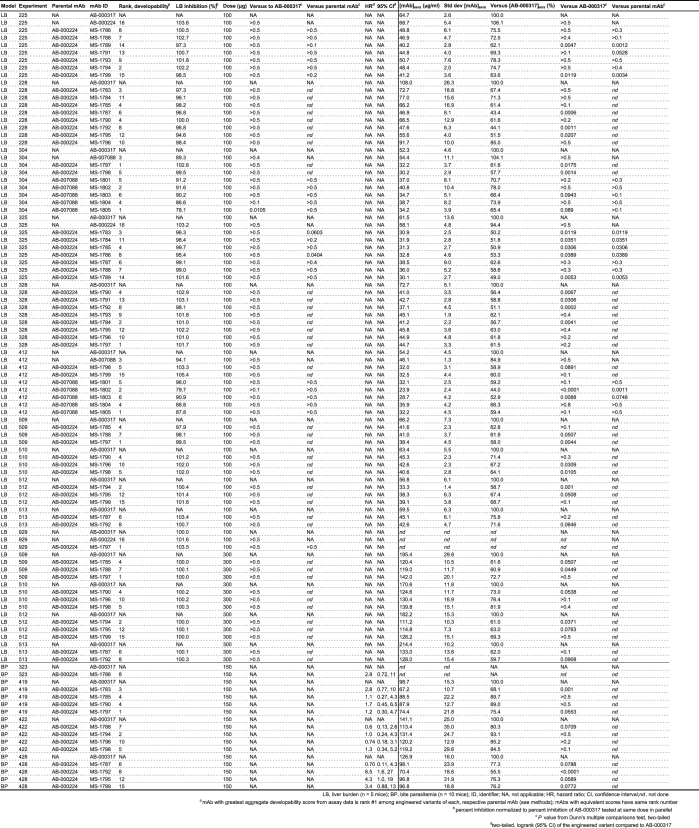
Developability ranking and in vivo pharmacology of engineered variant mAbs

## Data Availability

Accession numbers for all paired heavy- and light-chain IgG sequences recombinantly expressed were provided through BankIt: 2749610: OR662637–OR663656. The entire set of unique natively paired IgG sequences (*n* = 28,672) from PBs (*n* = 32,948) of RTS,S vaccines (*n* = 45) are available at https://zenodo.org/records/10019777. Requests for other datasets generated and/or analyzed in the current study will be promptly reviewed by the corresponding authors (malaria@biosimplify.com or kwilliams@atreca.com), and a Material Transfer Agreement (MTA) provided should the request be subject to intellectual property obligations. Materials subject to an MTA will be released pending execution. All other data/materials not subject to an MTA will be provided within a reasonable time frame following the initial request. Data from the clinical trial of RTS,S are reported elsewhere^24^. Source data are provided with this paper.

## Code availability

The custom code used in R for bootstrap analyses is available at https://github.com/expositum/RTS_S_CSPBinding_vs_Protection. Custom code used in the TitrationAnalysis tool for SPR analyses is available at https://github.com/DukeCHSI/TitrationAnalysis. All materials used in the study are commercially available except for PBMC samples obtained from the clinical trial (NCT01857869). Antibodies can be expressed as recombinant proteins via gene synthesis and expression using IgG sequence data and methods (see paired IgG sequence repertoire datasets (above) and ‘Recombinant antibody production’).
