# Supplementary material for: A candidate antibody drug for prevention of malaria
Source: Nat Med. 2024 Jan 2;30(1):117–29. doi: 10.1038/s41591-023-02659-z (PMC10803262; doi:10.1038/s41591-023-02659-z)
Supplement: Supplementary file 1 — Supplementary Figs. 1–9, including text legend for each. [file 41591_2023_2659_MOESM1_ESM.pdf]

nature medicine

Article https://doi.org/10.1038/s41591-023-02659-z

A candidate antibody drug for prevention of malaria

In the format provided by the authors and unedited

Supplementary Figures

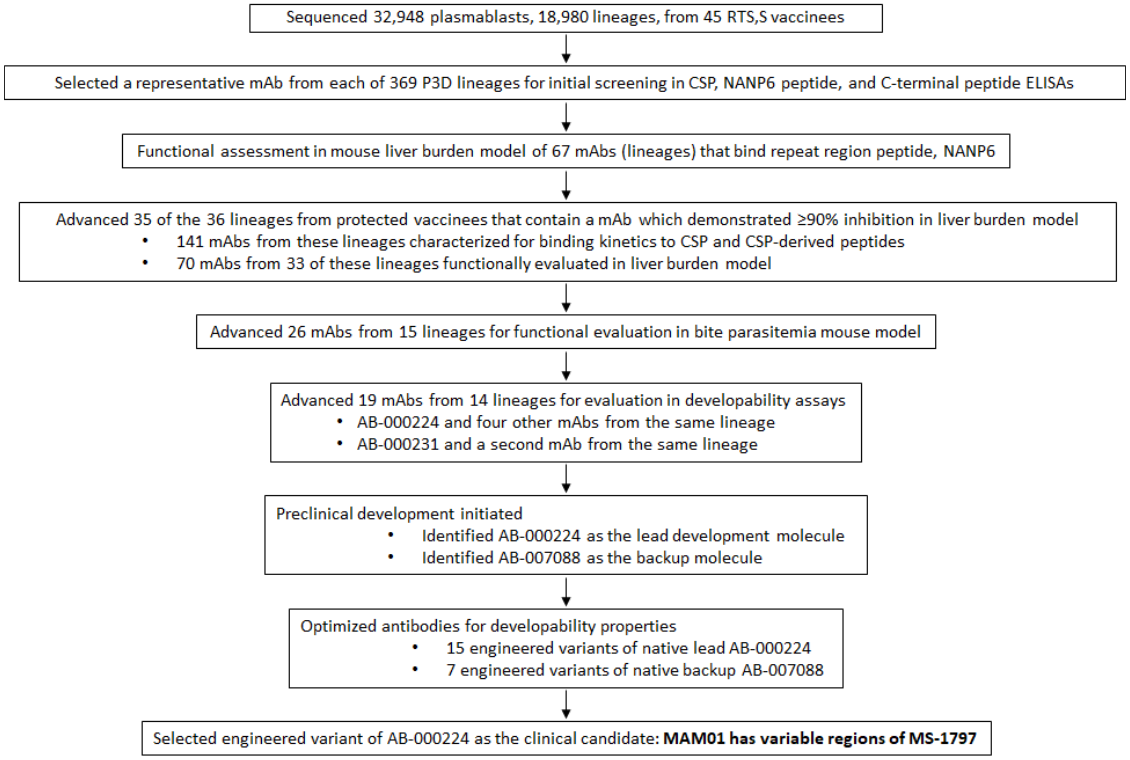

Supplementary Fig. 1 | Downselection steps from IgG sequence repertoires to engineered clinical candidate, MAM01. Flow chart summarizes steps by which IgG sequences from 32,948 plasmablasts were used to generate a screening library of 369 recombinant antibodies from which the lead antibody, AB-000224, was derived and further engineered to generate MAM01.

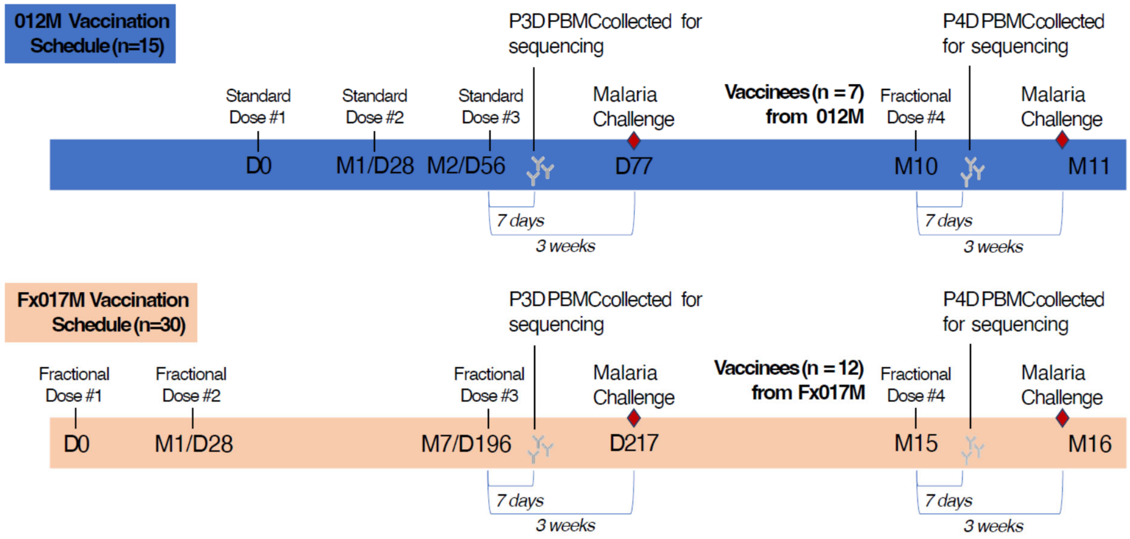

Supplementary Fig. 2 | Schedules of dose regimens, sample collections for PB sequencing, and malaria challenges from an RTS,S phase 2a clinical trial. The vaccination trial[24] compared vaccinees who received one of two dosing regimens of RTS,S/AS01 prior to controlled human malaria infection (CHMI). The standard dose group (012M, blue, n = 15) and the fractional dose group (Fx017M, orange, n = 30) are each represented by separate timelines with the number of days (D) and months (M) indicated in relation to administration of the first dose (not to scale). The fractional dose group received a delayed third dose that was one-fifth the volume of a standard dose (“fractional dose”). CHMI was performed approximately 3 weeks after the third dose. A subset of vaccinees from both dose groups (012M, n = 7; Fx017M, n = 12) received a fourth, fractional dose before a second CHMI the occurred approximately 8 months after the first one. PBMC were collected 7 days after the third (P3D) and the fourth (P4D) doses approximately two weeks prior to CHMI.

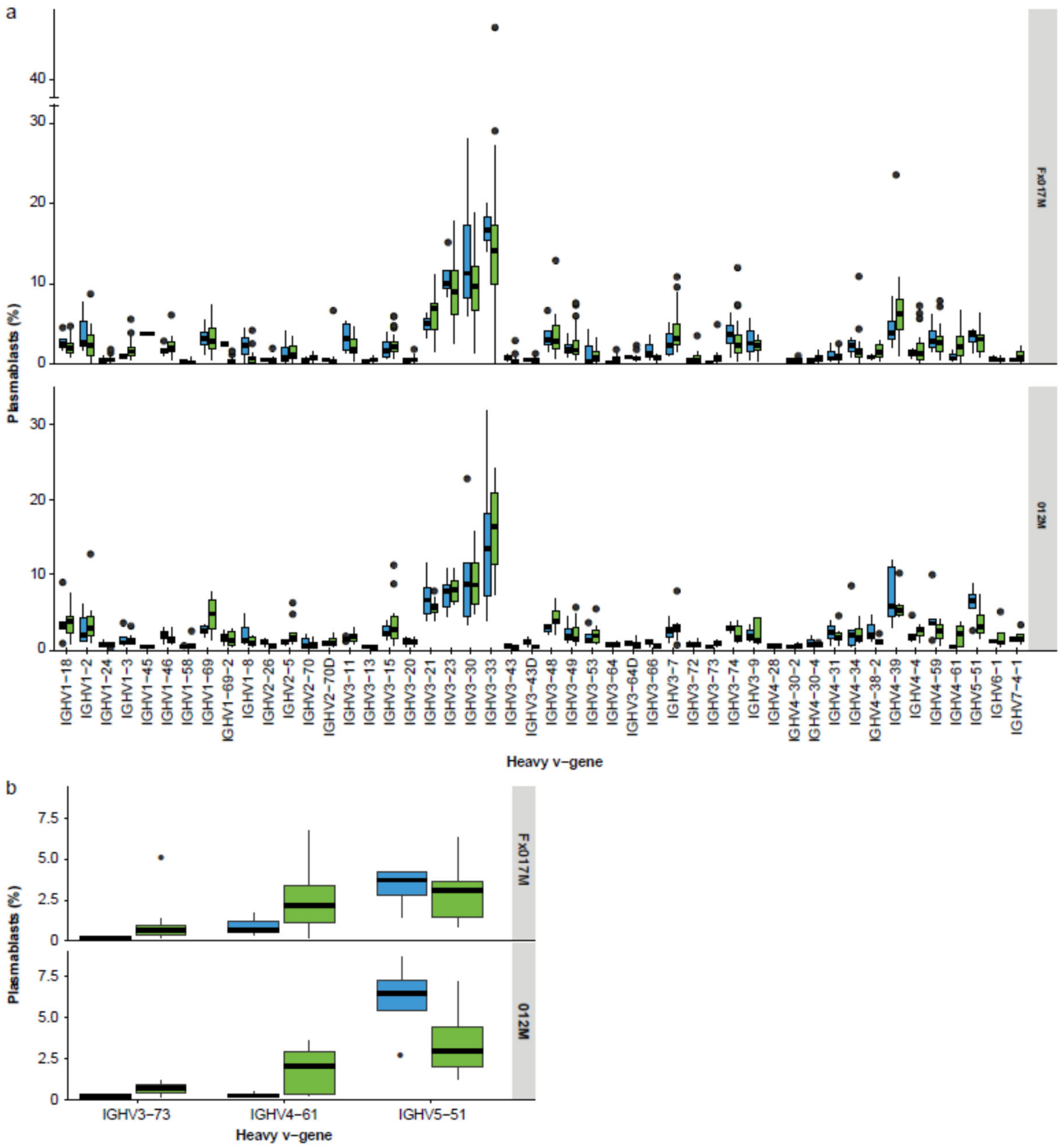

Supplementary Fig. 3 | IgG heavy chain germline gene usage from the PB response after the third dose of RTS,S. a–b, Germline V-gene usage in heavy chains, **a**, compared between protected (green, n = 36) and unprotected (blue, n = 9) vaccinees and across dose groups (standard dose group, “012M”, n = 15; fractional dose group, “Fx017M”, n = 30) with IGHV3-30 and IGHV3-33 showing high prevalence. **b**, Three heavy chain germline genes, IGHV3-73, IGHV4-61, and IGHV5-51, were initially associated with vaccinees’ protection status ($P < 0.05$, Wilcoxon Rank Sum test) but were not associated after correcting for mul-

tiple hypothesis testing. All were $P > 0.05$, Benjamini–Hochberg or Bonferroni tests. No significant associations were detected between vaccinees' protection status or dose groups. Boxes indicate interquartile ranges, lines within boxes are medians, whiskers represent farthest data points within 1.5 x the interquartile range, and points outside whiskers are plotted individually as outliers.

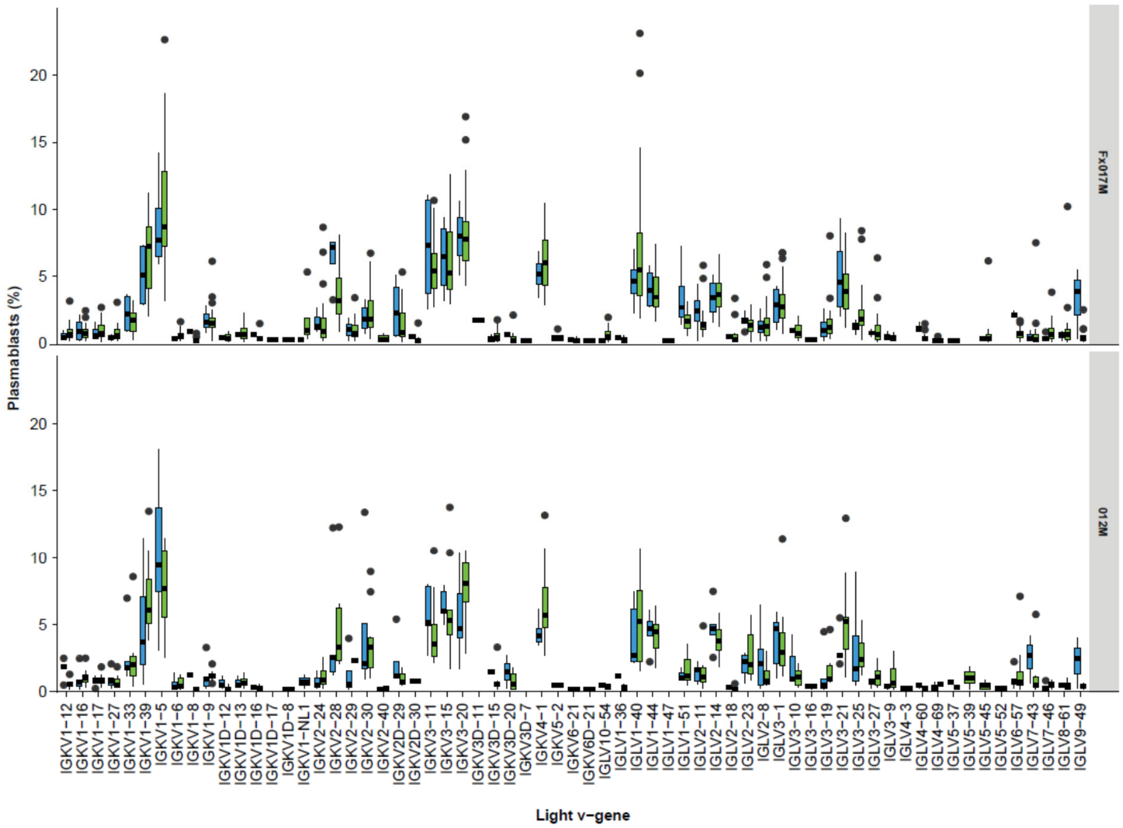

Supplementary Fig. 4 | IgG light chain germline gene usage from the PB response after the third dose of RTS,S. Germline V-gene usage in light chains compared between protected (green, n = 36) and unprotected (blue, n = 9) vaccinees and across dose groups (standard dose group, “012M”, n = 15; fractional dose group, “Fx017M”, n = 30) with KV1-5, KV3-20, and LV1-40 showing high prevalence. All were P > 0.05, Benjamini–Hochberg or Bonferroni tests. No significant associations were detected between vaccinees’ protection status or dose groups. Boxes indicate interquartile ranges, lines within boxes are medians, whiskers represent farthest data points within 1.5 x the interquartile range, and points outside whiskers are plotted individually as outliers.

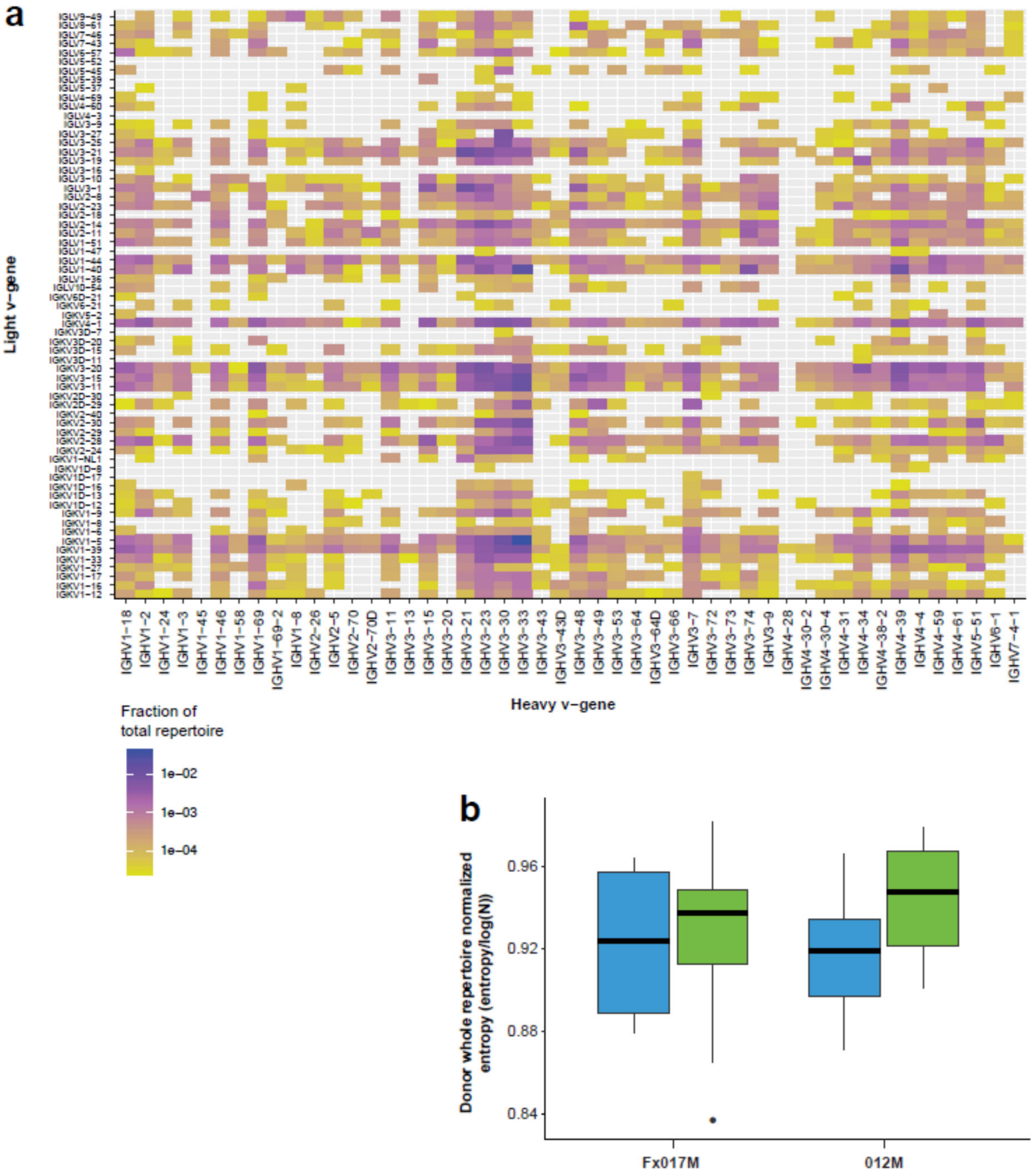

Supplementary Fig. 5 | Frequency of heavy and light chain V-gene pairings and clonality of IgG sequences from the PB response after the third dose of RTS,S. a, Heat map indicating the frequency of specific pairings of heavy and light chain V-genes among IgG sequences from all vaccinees (n = 45). **b**, Repertoire clonality for analyses that included the lineages that contain ≥1 PB compared between protected (green, n = 36) and unprotected (blue, n = 9) vaccinees and across dose groups (standard dose group, “012M”, n = 15; fractional

dose group, “Fx017M”, n = 30), Normalised Shannon entropy, P > 0.05 for all analyses (Wilcoxon rank sum test or Kolmogorov–Smirnov test). Boxes indicate interquartile ranges, lines within boxes are medians, whiskers represent farthest data points within 1.5 x the interquartile range, and points outside whiskers are plotted individually as outliers.

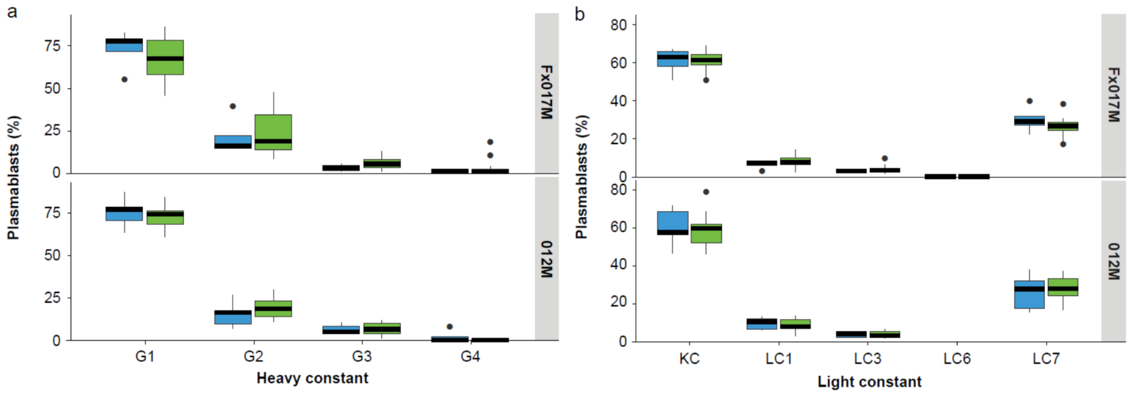

Supplementary Fig. 6 | Constant region subclass of IgG sequences from the PB response after the third dose of RTS,S. a–b, IgG isotype compared between protected (green, n = 36) and unprotected (blue, n = 9) vaccinees and across dose groups (standard dose group, “012M”, n = 15; fractional dose group, “Fx017M”, n = 30) for, **a**, heavy and, **b**, light chain constant region subclass (P > 0.05 for all analyses, Wilcoxon Rank Sum test). No significant associations were detected between vaccinees’ protection status or dose groups. Boxes indicate interquartile ranges, lines within boxes are medians, whiskers represent farthest data points within 1.5 x the interquartile range, and points outside whiskers are plotted individually as outliers.

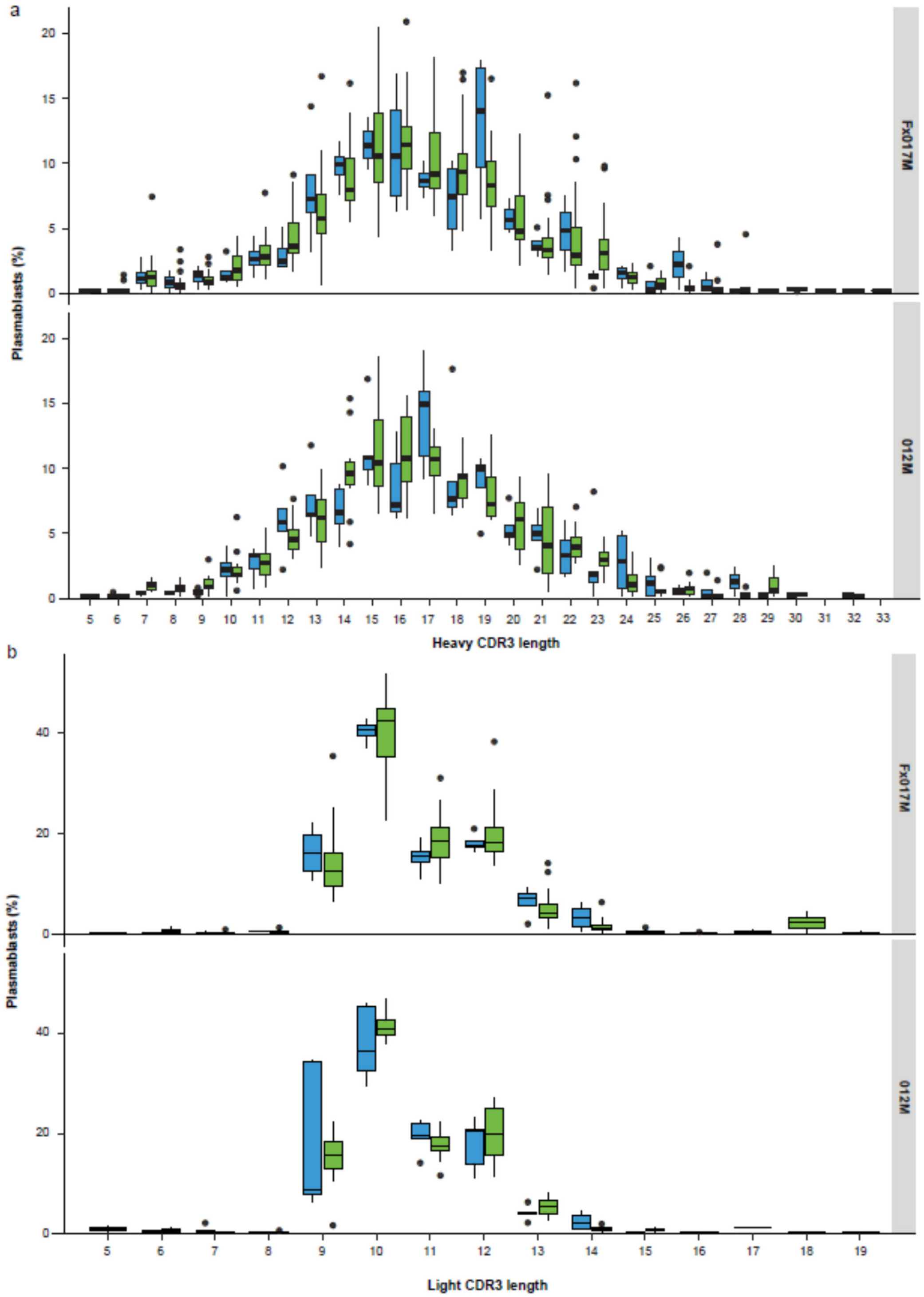

Supplementary Fig. 7 | Length of CDR3 regions of IgG sequences from the PB response after the third dose of RTS,S. a–b, Lengths of the complementarity determining region 3 (CDR3) compared between protected (green, n = 36) and unprotected (blue, n = 9) vaccinees

and across dose groups (standard dose group, “012M”, n = 15; fractional dose group, “Fx017M”, n = 30) for, **a**, heavy or, **b**, light chains ($P > 0.05$ for both, Wilcoxon Rank Sum test). No significant associations were detected between vaccinees’ protection status or dose groups. Boxes indicate interquartile ranges, lines within boxes are medians, whiskers represent farthest data points within 1.5 x the interquartile range, and points outside whiskers are plotted individually as outliers.

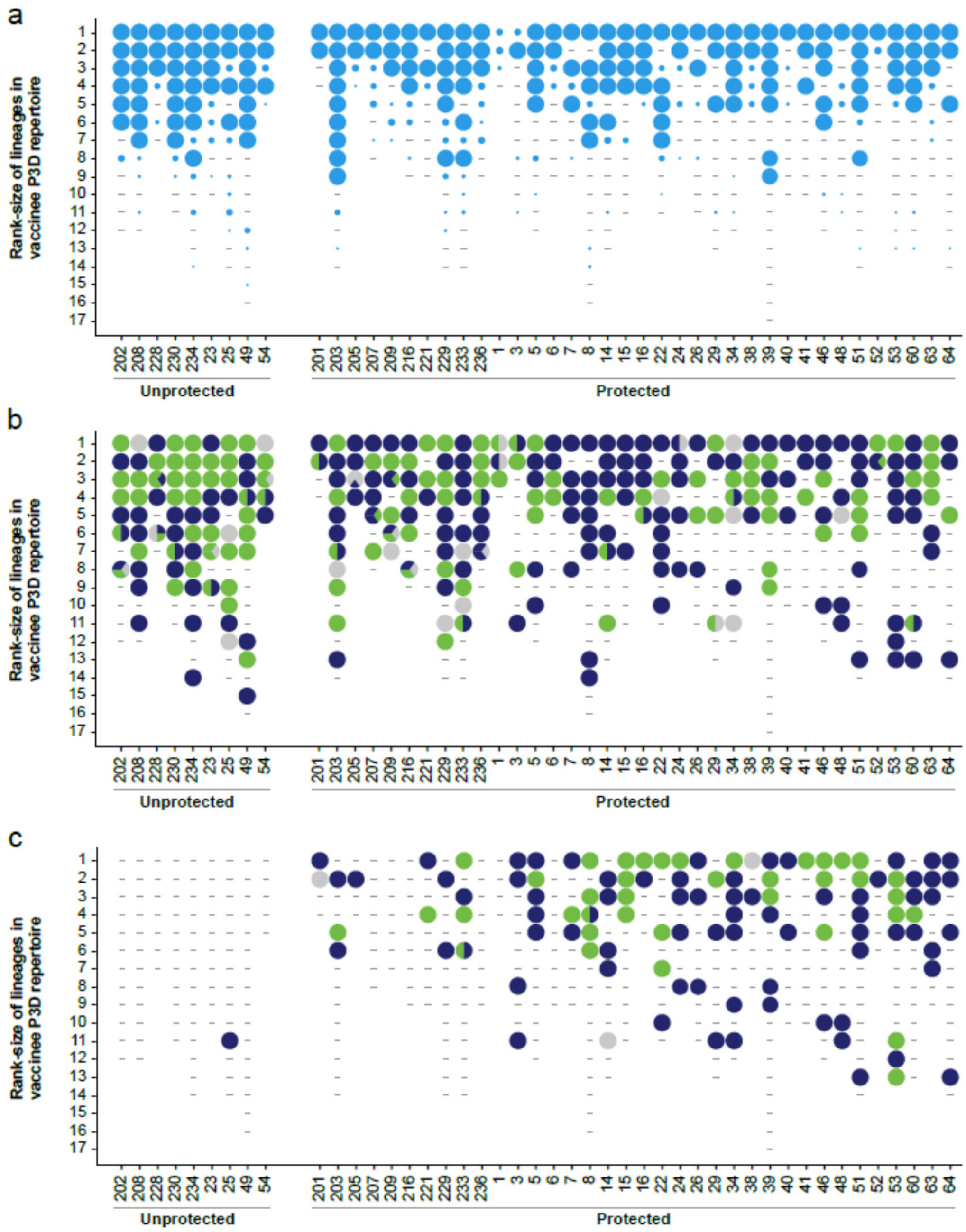

Supplementary Fig. 8 | Antibody lineages tested in binding assays and reactivity to CSP or HBsAg. a–c, Rank-size of expanded lineages in each protected and unprotected vaccinee repertoire from PBs collected 7 days after the third dose of RTS,S ("P3D"). **a**, Expanded PB antibody lineages (circles representing ≥1 lineages) for each rank-size and vaccinee from which a mAb was selected, recombinantly expressed and screened in the CSP ELISA (n = 349 mAbs, 282 circles). Lineages from which a mAb was tested in the CSP ELISA represent

8.9%-84% (median of 37%) of all PB in expanded lineages of vaccinee repertoires. Circle sizes are proportional to the fraction of lineages tested among all lineages observed at each rank-size and vaccinee. Lineages from a vaccinee that have the same number of PBs have the same rank-size. The largest circles indicate that all lineages from the vaccinee at that rank-size were tested. The smallest circle indicates only one 1 of the 58 lineages observed from the vaccinee at that rank-size was tested. In some cases, none of the lineages from a vaccinee at an indicated rank-size were tested (grey bars). **b–c**, Expanded PB antibody lineages (circles representing 1–5 lineages) for each rank-size and vaccinee from which a mAb was tested in, **b**, the CSP ELISA, reactive (green, n = 135 mAbs, 94 circles), indeterminant (grey, n = 29 mAbs, 14 circles), not reactive (blue, n = 185 mAbs, 144 circles), or a combination of these outcomes for different mAbs from lineages of same rank-size and vaccinee (pie charts of mixed colours, 30 circles), or tested in, **c**, the HBsAg ELISA, reactive (green, n = 38 mAbs, 36 circles), indeterminant (grey, n = 3 mAbs, 3 circles), not reactive (blue, n = 77 mAbs, 72 circles), or a combination of these outcomes for different mAbs from lineages of same rank-size and vaccinee (pie charts of mixed colours, 2 circles).

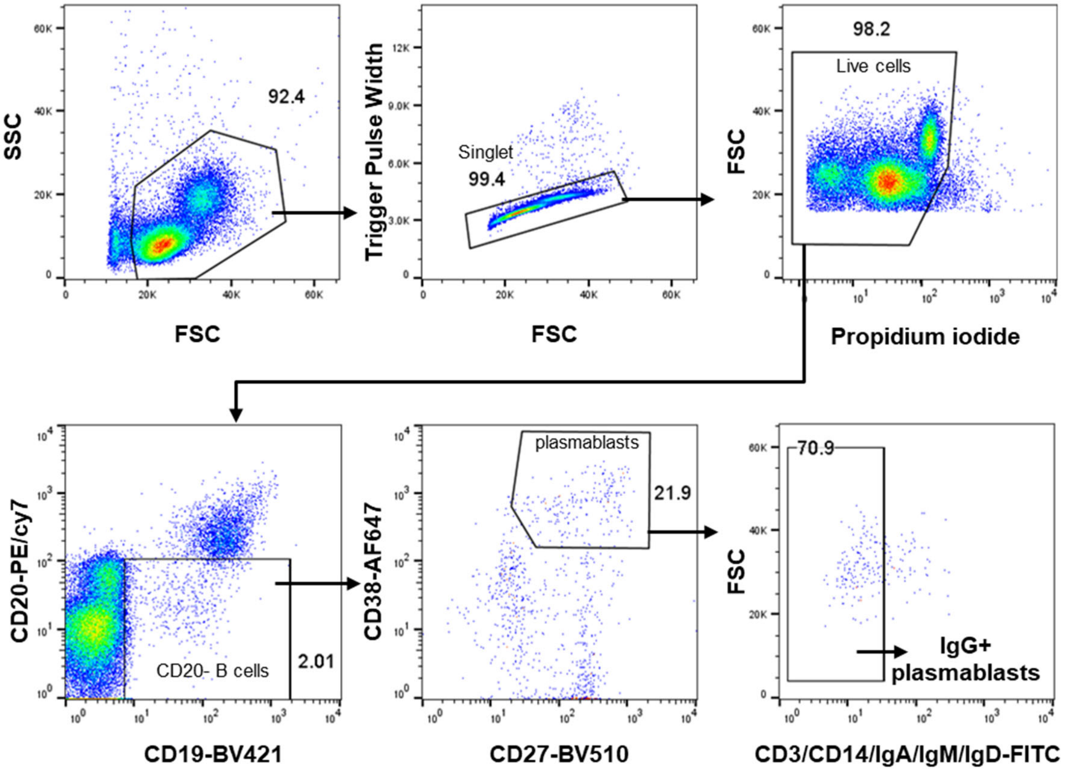

Supplementary Fig. 9 | Isolation of plasmablasts by fluorescence-activated cell sorting

Flow cytometric identification of plasmablasts collected as CD3−CD14−CD19+CD20−CD27+CD38++IgA−IgM−IgD− cells from a PBMC sample of an RTS,S vaccinee (Fx017M) prior to IgG sequencing. The cell type isolated by the gate is indicated and numbers represent the percentage of events within the gate.
