# Supplementary material for: A candidate antibody drug for prevention of malaria
Source: Nat Med. 2024 Jan 2;30(1):117–29. doi: 10.1038/s41591-023-02659-z (PMC10803262; doi:10.1038/s41591-023-02659-z)
Supplement: Supplementary file 2 — Reporting Summary [file 41591_2023_2659_MOESM2_ESM.pdf]

nature portfolio

Corresponding author(s): Katherine L Williams
Daniel E Emerling

Last updated by author(s): Oct 10, 2023

Reporting Summary

Nature Portfolio wishes to improve the reproducibility of the work that we publish. This form provides structure for consistency and transparency in reporting. For further information on Nature Portfolio policies, see our Editorial Policies and the Editorial Policy Checklist.

Statistics

For all statistical analyses, confirm that the following items are present in the figure legend, table legend, main text, or Methods section.

n/a	Confirmed	
☐	☒	The exact sample size (*n*) for each experimental group/condition, given as a discrete number and unit of measurement
☐	☒	A statement on whether measurements were taken from distinct samples or whether the same sample was measured repeatedly
☐	☒	The statistical test(s) used AND whether they are one- or two-sided<br>*Only common tests should be described solely by name; describe more complex techniques in the Methods section.*
☐	☒	A description of all covariates tested
☐	☒	A description of any assumptions or corrections, such as tests of normality and adjustment for multiple comparisons
☐	☒	A full description of the statistical parameters including central tendency (e.g. means) or other basic estimates (e.g. regression coefficient) AND variation (e.g. standard deviation) or associated estimates of uncertainty (e.g. confidence intervals)
☐	☒	For null hypothesis testing, the test statistic (e.g. *F*, *t*, *r*) with confidence intervals, effect sizes, degrees of freedom and *P* value noted<br>*Give P values as exact values whenever suitable.*
☒	☐	For Bayesian analysis, information on the choice of priors and Markov chain Monte Carlo settings
☒	☐	For hierarchical and complex designs, identification of the appropriate level for tests and full reporting of outcomes
☒	☐	Estimates of effect sizes (e.g. Cohen's *d*, Pearson's *r*), indicating how they were calculated

Our web collection on statistics for biologists contains articles on many of the points above.

Software and code

Policy information about availability of computer code

Data collection

Plasmablasts were collected using BD FACSDiva software v8.0.3
SPR binding kinetic data was collected on the Carterra LSA using Epitope Software v1.5.
Next-gen sequences were collected using Roche GS FLX+ System Software v2.9
In vivo luminescence data was collected using Living Image v3.2 software (Perkin Elmer)
ELISA data were collected using Biotek Gen5 software v2 or Molecular Devices microplate reader with SoftMax-Pro GxP version 6.5.1

Data analysis

The kinetics titration data collected were first pre-processed in the NextGenKIT (Carterra) software v1.7. The data were then exported and analysed using the TitrationAnalysis tool (https://zenodo.org/record /7998652). Custom code for the tool available at https://github.com/DukeCHSI/TitrationAnalysis.

In vivo luminescence data were analyzed using Living Image v3.2 software (Perkin Elmer)

All statistical tests were performed using GraphPad Prism v8 or v9 except for the Bootstrap analyses and the Benjamini-Hochberg False Discovery Rate analysis which were performed using R v3.4.4 (cran.r-project.org).
Custom code used in R for bootstrap analyses is available at the site https://github.com/expositum/Mal71_CSPBinding_vs_Protection.
Custom code used in the TitrationAnalysis tool for SPR analyses is available at https://github.com/DukeCHSI/TitrationAnalysis

Cell counts for engineered production cell lines were analysed using Guava CytoSoft Data Acquisition and Analysis Software v3.2

ELISA data were analyzed using Biotek Gen5 software v2

IgG sequences were assembled to variable (V), diversity (D) and joining (J) gene segment assignments and somatic hypermutations (SHM) were identified using Somatic Diversification Analysis [SoDA, Volpe, J. M., Cowell, L. G. & Kepler, T. B. SoDA: implementation of a 3D alignment algorithm for inference of antigen receptor recombinations. Bioinformatics 22, 438–444 (2006)] and the IMGT human immunoglobulin germline database release, IMGT_202031
Structural models were made using Molecular Operating Environment, MOE, Molecular Operating Environment, 2022.02 Chemical Computing Group ULC

Other analyses were performed using Microsoft Excel (multiple versions)

For manuscripts utilizing custom algorithms or software that are central to the research but not yet described in published literature, software must be made available to editors and reviewers. We strongly encourage code deposition in a community repository (e.g. GitHub). See the Nature Portfolio guidelines for submitting code & software for further information.

Data

Policy information about availability of data

All manuscripts must include a data availability statement. This statement should provide the following information, where applicable:

- Accession codes, unique identifiers, or web links for publicly available datasets
- A description of any restrictions on data availability
- For clinical datasets or third party data, please ensure that the statement adheres to our policy

IgG sequence datasets Accession numbers for all paired heavy and light chain IgG sequences that were recombinantly expressed were provided through BankIt: 2749610: OR662637 - OR663656). The entire set of unique natively paired IgG sequences (n = 28,672) from PBs (n = 32,948) of RTS,S vaccines (n = 45) are available at the site, https://zenodo.org/record/8436761.

Requests for other datasets generated and/or analysed in the current study will be promptly reviewed by the corresponding authors (emerling@biosimplify.com or kwilliams@atreca.com) and a Material Transfer Agreement provided should the request be subject to intellectual property obligations. Materials subject to an MTA will be released pending execution. All other data/ materials not subject to an MTA will be provided within a reasonable timeframe following the initial request.

Human research participants

Policy information about studies involving human research participants and Sex and Gender in Research.

Reporting on sex and gender	Previously reported in Regules, J. A. et al. Fractional third and fourth dose of RTS,S/AS01 malaria candidate vaccine: A phase 2a controlled human malaria parasite infection and immunogenicity study. J. Infect. Dis. 214, 762–771 (2016)]<br><br>The ratio of females to males was 15:19 and 6:11 in the Fx017M, 012M groups respectively.
Population characteristics	Previously reported in Regules, J. A. et al. Fractional third and fourth dose of RTS,S/AS01 malaria candidate vaccine: A phase 2a controlled human malaria parasite infection and immunogenicity study. J. Infect. Dis. 214, 762–771 (2016)]<br><br>Vaccinees included males and non-pregnant females aged 18–50 years who were free of any serious acute or chronic illness, as determined by clinical or physical examination, medical history records, or laboratory screening tests of hematologic, renal, and hepatic function; did not have a history of malaria; were seronegative for HBsAg, hepatitis C virus, and human immunodeficiency virus (HIV); and had the ability to comply with the study protocol.<br>The mean age of subjects was 33.6 years.<br>The ratio of females to males was 15:19 and 6:11 in the Fx017M, 012M groups respectively.
Recruitment	Previously reported in Regules, J. A. et al. Fractional third and fourth dose of RTS,S/AS01 malaria candidate vaccine: A phase 2a controlled human malaria parasite infection and immunogenicity study. J. Infect. Dis. 214, 762–771 (2016)]<br><br>The target enrollment was 65 volunteers with consecutive (nonrandomized and open) allocation to study groups: the first 34 volunteers were to immunized according to a 0-, 1-, and 7-month schedule with a fractional third dose (Fx017M), the subsequent 17 volunteers were immunized with 3 full doses according to a 0-, 1-, and 2-month schedule (012M). For this study, we received PBMC samples from 30 of the 017M vaccinees and 15 of the 012M vaccinees.
Ethics oversight	The protocol was approved by the Walter Reed Army Institute of Research Institutional Review Board and the Western Institutional Review Board, and written informed consent was obtained from each subject before study procedures were initiated (ClinicalTrials.gov identifier: NCT01857869 and Regules, J. A. et al. Fractional third and fourth dose of RTS,S/AS01 malaria candidate vaccine: A phase 2a controlled human malaria parasite infection and immunogenicity study. J. Infect. Dis. 214, 762–771 (2016)]

Note that full information on the approval of the study protocol must also be provided in the manuscript.

Field-specific reporting

Please select the one below that is the best fit for your research. If you are not sure, read the appropriate sections before making your selection.

☒ Life sciences ☐ Behavioural & social sciences ☐ Ecological, evolutionary & environmental sciences

Life sciences study design

All studies must disclose on these points even when the disclosure is negative.

Sample size

The main aim of this study was to downselect an antibody clone for potential development as a drug from sequence repertoires of RTS,S vaccinees. Sample size of vaccinees sequenced was predetermined based on the RTS,S clinical trial study protocol and the PBMC samples obtained from that trial. The sample size of plasmablasts collected for sequencing was based on the exhaustive use of the samples to obtain plasmablasts. The number of antibody lineages included in the first screen for discovery of CSP-binding antibodies was determined based on goal to analyze a majority of the most dominant lineages from all the vaccinees. The sample size of antibodies selected for functional tests was based on the limited throughput of animal model functional testing.

Technical replicates used in the initial antigen binding screens were duplicates for each antibody, and were duplicates or triplicates for the . SPR binding experiments. In all these binding assays, standard replicate numbers were used as common in the field and no statistical determination was made for technical replicate numbers used. For SPR binding kinetics determination, the number of concentrations was determined by making a two-fold dilution series that provided enough data points to determine Koff, Kon, and KD values.

For in vivo studies, each antibody was tested in 5 mice (liver burden model) or 10 mice (mosquito bite parasitemia model) as a sample size that minimizes the use of animals while still providing a means to statistically compare the activity of test articles to control conditions as reported elsewhere [Flores-Garcia, Y. et al. Optimization of an in vivo model to study immunity to Plasmodium falciparum pre-erythrocytic stages. Malar. J. 18, 426 (2019)].

Correlation analyses of sequence repertoire features, of binding data and protection status of vaccinees, of binding kinetics data and mutation (SHM) level data, of binding kinetics data and functional data, and of mutation (SHM) level data and functional data were all performed post-hoc with the available data that was obtained with the experimental goal of screening for, discovering and engineering an anti-CSP drug. Thus, no power analyses were performed to determine sample size for these correlation analyses prior to experimental design, however sample size would not appear limiting for those cases in which a statistically significant correlation or inverse-correlation was reported.

Data exclusions

No animals or data points were excluded from the analyses of animal model data.

For SPR binding experiments involving the engineered variants, some kinetic data were excluded from KD calculations per predetermined, data quality, acceptance criteria: i) standard error of the estimated kon, koff and KD in each replicate ≤20% and ii) fold-change for all three parameters within the triplicate ≤3.

Replication

In cases where antibodies from vaccinees' repertoires were tested in CSP or peptide ELISA assays more than once, reproduced positive, negative, or indeterminate results (see Methods) were obtained for CSP, NANP6, or C-terminal binding.

Results from other binding experiments are reported as mean values from all replicates. Reproducibility was based on technical replicates as described in the "Sample size" section above.

For some in vivo functional assessments of particular antibodies, independent experiments were reproduced and outcomes of these reproductions are reported in the manuscript.

Randomization

Enrollment and participation in the RTS,S clinical trial was as previously reported in Regules, J. A. et al. Fractional third and fourth dose of RTS,S/AS01 malaria candidate vaccine: A phase 2a controlled human malaria parasite infection and immunogenicity study. J. Infect. Dis. 214, 762–771 (2016)].
The target enrollment was 65 volunteers with consecutive (nonrandomized and open) allocation to study groups: the first 34 volunteers were to immunized according to a 0-, 1-, and 7-month schedule with a fractional third dose (Fx017M), the subsequent 17 volunteers were immunized with 3 full doses according to a 0-, 1-, and 2-month schedule (012M). For this study, we received PBMC samples from 30 of the 017M vaccinees and 15 of the 012M vaccinees.

Randomization of mice was not needed per protocols of the malaria models used.

Randomization of PBMC samples and antibodies was not appropriate/relevant to the study

Blinding

Linkage of vaccine trial participant information (i.e. protection status and trial arm assignment) and PBMC samples, IgG sequences derived from the PBMC, and recombinant antibodies derived from the sequences was blinded to those researchers who isolated plasmablasts, sequenced IgG, and tested antibodies in initial binding and in vivo functional screens.

Reporting for specific materials, systems and methods

We require information from authors about some types of materials, experimental systems and methods used in many studies. Here, indicate whether each material, system or method listed is relevant to your study. If you are not sure if a list item applies to your research, read the appropriate section before selecting a response.

Materials & experimental systems

n/a	Involved in the study
☐	☒ Antibodies
☐	☒ Eukaryotic cell lines
☒	☐ Palaeontology and archaeology
☐	☒ Animals and other organisms
☐	☒ Clinical data
☒	☐ Dual use research of concern

Methods

n/a	Involved in the study
☒	☐ ChIP-seq
☐	☒ Flow cytometry
☒	☐ MRI-based neuroimaging

Antibodies

Antibodies used

anti-CD3-FITC (BioLegend, cat# 300440, clone UCHT1, 1:100), anti-CD14-FITC (Bio-Legend, cat# 325604, clone HCD14, 1:100), anti-CD19-BV421 (BioLegend, cat# 302234, clone HIB19, 1:100), anti-CD20-PE/cy7 (Biolegend, cat# 302312, clone 2H7, 1:100), anti-CD27-BV510 (BioLegend, cat# 302836, clone O323, 1:50), anti-CD38-A647 (BioLegend, cat# 303514, clone HIT2, 1:100), anti-IgA-FITC (Miltenyi, cat# 130- 113-475, clone IS11-8E10, 1:50), anti-IgM-FITC (BioLegend, cat# 314506, clone MHM-88, 1:50), anti-IgD-FITC (Biolegend #348206, clone IA6-2, 1:50)

Monoclonal antibodies discovered and characterized in the study are defined by their heavy and light chain variable regions' sequences and can be gene synthesized and recombinantly expressed from those sequences which will be provided via a public data repository (please see availability of data statement)

Validation

https://www.biolegend.com/en-us/products/fitc-anti-human-cd3-antibody-863?pdf=true&displayInline=true&leftRightMargin=15&topBottomMargin=15&filename=FITC%20anti-human%20CD3%20Antibody.pdf&v=20220902063018

https://www.biolegend.com/en-us/products/fitc-anti-human-cd14-antibody-3951?pdf=true&displayInline=true&leftRightMargin=15&topBottomMargin=15&filename=FITC%20anti-human%20CD14%20Antibody.pdf&v=20230114013553

https://www.biolegend.com/en-us/products/brilliant-violet-421-anti-human-cd19-antibody-7144?pdf=true&displayInline=true&leftRightMargin=15&topBottomMargin=15&filename=Brilliant%20Violet%20421%E2%84%A2%20anti-human%20CD19%20Antibody.pdf&v=20230114013553

https://www.bdbiosciences.com/content/bdb/paths/generate-tds-document.us.340955.pdf

https://www.biolegend.com/en-us/products/brilliant-violet-510-anti-human-cd27-antibody-8005?pdf=true&displayInline=true&leftRightMargin=15&topBottomMargin=15&filename=Brilliant%20Violet%20510%E2%84%A2%20anti-human%20CD27%20Antibody.pdf&v=20220831123135

https://www.biolegend.com/en-us/products/pe-cyanine7-anti-human-cd38-antibody-8420?pdf=true&displayInline=true&leftRightMargin=15&topBottomMargin=15&filename=PE/Cyanine7%20anti-human%20CD38%20Antibody.pdf&v=20230114013553

https://www.miltenyibiotec.com/US-en/products/iga-antibody-anti-human-is11-8e10.html#fitc:100-tests-in-200-ul

https://www.biolegend.com/en-us/products/apc-cyanine7-anti-human-igm-antibody-7403?pdf=true&displayInline=true&leftRightMargin=15&topBottomMargin=15&filename=APC/Cyanine7%20anti-human%20IgM%20Antibody.pdf&v=20220817065308

https://d1spbj2x7qk4bg.cloudfront.net/ja-jp/products/fitc-anti-human-igd-antibody-6683?pdf=true&displayInline=true&leftRightMargin=15&topBottomMargin=15&filename=FITC%20anti-human%20IgD%20Antibody.pdf&v=20230714033116

Eukaryotic cell lines

Policy information about cell lines and Sex and Gender in Research

Cell line source(s)

HEK293 cells (ATCC)
CHO-K1 GS KO cells (Horizon Discovery)

Authentication

Authentication via certificate of authentication from supplier

Mycoplasma contamination

Production cell line, CHO-K1, was tested for contamination per FDA guidelines and requirements

Commonly misidentified lines
(See ICLAC register)

Name any commonly misidentified cell lines used in the study and provide a rationale for their use.

Animals and other research organisms

Policy information about studies involving animals; ARRIVE guidelines recommended for reporting animal research, and Sex and Gender in Research

Laboratory animals	Studies using mice were carried out using 6-8 weeks old C57BL/6 female mice (Charles River Labs), maintained at the animal facility of the Johns Hopkins Bloomberg School of Public Health. The assays using mice were performed in strict accordance with the recommendations in the Guide for the Care and Use of Laboratory Animals of the National Institutes of Health. The protocol was approved by the Animal Care and Use Committee of the Johns Hopkins University, protocol numbers, MO18H419 and MO21H417. No animals or data points were excluded from the analyses<br><br>Approximately 3000 mice were used across the in vivo experiments reported in this study.
Wild animals	The study did not involve wild animals
Reporting on sex	All experiments were done using female mice. Our studies have shown that female mice are more susceptible to infection*. Thus, the infection assays have a dynamic range that allow us to differentiate better between antibodies with different protective efficacy.<br><br>* Flores-Garcia et al., Malaria Journal volume 18, Article number: 426 (2019)<br>Optimization of an in vivo model to study immunity to Plasmodium falciparum pre-erythrocytic stages
Field-collected samples	The study did not involve samples collected from the field
Ethics oversight	The assays using mice were performed in strict accordance with the recommendations in the Guide for the Care and Use of Laboratory Animals of the National Institutes of Health. The protocol was approved by the Animal Care and Use Committee of the Johns Hopkins University, protocols number MO18H419 and MO21H417

Note that full information on the approval of the study protocol must also be provided in the manuscript.

Clinical data

Policy information about clinical studies

All manuscripts should comply with the ICMJE guidelines for publication of clinical research and a completed CONSORT checklist must be included with all submissions.

Clinical trial registration	ClinicalTrials.gov identifier: NCT01857869<br>Human research participants were part of the phase 2a clinical trial of RTS,S/AS01B which has been reported previously [Regules, J. A. et al. Fractional third and fourth dose of RTS,S/AS01 malaria candidate vaccine: A phase 2a controlled human malaria parasite infection and immunogenicity study. J. Infect. Dis. 214, 762–771 (2016)]. The protocol was approved by the Walter Reed Army Institute of Research Institutional Review Board and the Western Institutional Review Board, and written informed consent was obtained from each subject before study procedures were initiated
Study protocol	Reported previously in Regules, J. A. et al. Fractional third and fourth dose of RTS,S/AS01 malaria candidate vaccine: A phase 2a controlled human malaria parasite infection and immunogenicity study. J. Infect. Dis. 214, 762–771 (2016)].
Data collection	Reported previously in Regules, J. A. et al. Fractional third and fourth dose of RTS,S/AS01 malaria candidate vaccine: A phase 2a controlled human malaria parasite infection and immunogenicity study. J. Infect. Dis. 214, 762–771 (2016)].
Outcomes	Reported previously in Regules, J. A. et al. Fractional third and fourth dose of RTS,S/AS01 malaria candidate vaccine: A phase 2a controlled human malaria parasite infection and immunogenicity study. J. Infect. Dis. 214, 762–771 (2016)].

Flow Cytometry

Plots

Confirm that:

☒ The axis labels state the marker and fluorochrome used (e.g. CD4-FITC).

☒ The axis scales are clearly visible. Include numbers along axes only for bottom left plot of group (a 'group' is an analysis of identical markers).

☒ All plots are contour plots with outliers or pseudocolor plots.

☒ A numerical value for number of cells or percentage (with statistics) is provided.

Methodology

Sample preparation	Plasmablast cells were isolated/collected by flow cytometry and used for sequencing of IgG. Gates are shown in Supplementary Fig. 9 with percentage of cells within each gate noted (statistics are not applicable)<br><br>Plasmablast isolation, cloning, and sequencing were performed using BD FACSDiva soft-ware v8.0.3 and the previously published protocol78 with the following modifications. PBMC were stained with the following mAbs and dilutions: anti-CD3-FITC (BioLegend, cat# 300406, clone UCHT1, 1:100), anti-CD14-FITC (Bio-Legend, cat# 325604, clone HCD14, 1:100), anti-

	CD19-BV421 (BioLegend, cat# 302234, clone HIB19, 1:100), anti-CD20-PerCP/cy5.5 (BD, cat# 340955, clone L27, 1:15), anti-CD27-BV510 (BioLegend, cat# 302836, clone O323, 1:50), anti-CD38-PE/cy7 (BioLegend, cat# 356607, clone HB-7, 1:200), anti-IgA-FITC (Miltenyi, cat# 130- 113-475, clone IS11-8E10, 1:50), anti-IgM-APC/cy7 (BioLegend, cat# 314520, clone MHM-88, 1:100)
Instrument	BD FACSAria II
Software	BD FACSDiva software, v8.0.3
Cell population abundance	Purity of post-sort fractions could not be assessed in this study because IgG+ PBs were directly sorted one-cell-per-well into 96-well PCR plates containing hypotonic buffer that lysed the cells as intended for post-sort barcoding and sequening. Levels of target plasmablast cells among B cells and PBMC are reported in Supplementary Table 1
Gating strategy	Gating included both lymphoid and myeloid populations and excluded cell debris and aggregates in the FSC/SSC plot. Doublets and PI+ dead cells were gated out from the lymphoid and myeloid populations. Plasmablast target cells were then gated as the CD3-CD14-CD19+CD20−CD27+CD38++IgA−IgM− cells

☒ Tick this box to confirm that a figure exemplifying the gating strategy is provided in the Supplementary Information.
